# Supplementary material for: From Race to Racism: Teaching a Tool to Critically Appraise the Use of Race in Medical Research
Source: MedEdPORTAL. 2022 Jan 24;18:11210. doi: 10.15766/mep_2374-8265.11210 (PMC8784584; doi:10.15766/mep_2374-8265.11210)
Supplement: Supplementary file 1 — CARMeL Tool.docxCARMeL Workshop.pptxFacilitator Guide.docxParticipant Guide.docxUME Postsession Assessment.docxGME Pre- and Postsession Survey.docx [file mep_2374-8265.11210-s001.zip › E. UME Postsession Assessment.docx]

**Appendix E**

Undergraduate Medical Education Postsession Assessment

Please rate this session with regards to the following:

|  | **Unacceptable** | **Acceptable** | **Neutral** | **Good** | **Excellent** |
| --- | --- | --- | --- | --- | --- |
| Clarity of presentation | 1 | 2 | 3 | 4 | 5 |
| Quality of teaching | 1 | 2 | 3 | 4 | 5 |
| Quality of slides | 1 | 2 | 3 | 4 | 5 |
