# Supplementary material for: From Race to Racism: Teaching a Tool to Critically Appraise the Use of Race in Medical Research
Source: MedEdPORTAL. 2022 Jan 24;18:11210. doi: 10.15766/mep_2374-8265.11210 (PMC8784584; doi:10.15766/mep_2374-8265.11210)
Supplement: Supplementary file 1 — CARMeL Tool.docxCARMeL Workshop.pptxFacilitator Guide.docxParticipant Guide.docxUME Postsession Assessment.docxGME Pre- and Postsession Survey.docx [file mep_2374-8265.11210-s001.zip › F. GME Pre- and Postsession Survey.docx]

**Appendix F**

GME Pre- and Postsession Survey

| **Statement** | **Completely Disagree** | **Disagree** | **Neutral** | **Agree** | **Completely Agree** |
| --- | --- | --- | --- | --- | --- |
| I can differentiate concepts of race and ancestry as they apply to clinical practice | 1 | 2 | 3 | 4 | 5 |
| I can describe common flaws with the use of race as a biologic construct | 1 | 2 | 3 | 4 | 5 |
| I am able to appraise an article’s use of race | 1 | 2 | 3 | 4 | 5 |
