# Supplementary material for: From Race to Racism: Teaching a Tool to Critically Appraise the Use of Race in Medical Research
Source: MedEdPORTAL. 2022 Jan 24;18:11210. doi: 10.15766/mep_2374-8265.11210 (PMC8784584; doi:10.15766/mep_2374-8265.11210)
Supplement: Supplementary file 1 — CARMeL Tool.docxCARMeL Workshop.pptxFacilitator Guide.docxParticipant Guide.docxUME Postsession Assessment.docxGME Pre- and Postsession Survey.docx [file mep_2374-8265.11210-s001.zip › A. CARMeL Tool.docx]

**Appendix A: The Critical Appraisal of Race in Medical Literature (CARMeL) Tool**

| **Domain** | **Appraisal Questions** |
| --- | --- |
| **Internal Validity** | Do the authors clearly define race?  If so how? Is this definition consistent throughout the data collection, analysis and discussion? |
|  | To what extent does this article relay a biologic versus sociopolitical understanding of race? |
|  | To what extent do the authors clearly define how data on race were collected and organized? |
|  | If applicable, were those who analyzed race blinded to the trial interventions? |
| **External Validity** | To what extent do the options for race collected, reported and analyzed in this paper reflect typical, contemporary racial identities, or the understanding of racial identity with my patient population? |
|  | Are racial categories missing and/or conflated? |
| **Applicability/Impact** | Are there significant social, political or economic drivers of health that may be obscured by conclusions made in this article? |
|  | In what ways does the use of race in this article contribute to dominant narratives? |
| **Summary with Recommendation** | **CHOOSE ONE**:  *APPLY*: Race is used as a sociopolitical construct with appropriate methods and no significant threats to internal or external validity. The sociopolitical implications are evaluated and the data is deemed appropriate to apply to patients or populations.  *MODIFY*: Race is used as a sociopolitical construct, but with some threats to internal or external validity. In this case appropriate observations are accepted, but due to methodologic flaws providers will need to modify the way they are applied.  *CHALLENGE:* Race is likely used as a biologic construct, but with few to little other threats to the internal validity of the study. These studies often yield accurate observations of racial inequities in health, but draw inaccurate conclusions regarding causality (i.e. posit racial differences in the prevalence of illness or response to treatment, as opposed to racism and oppression as the cause of the observations). The results of such studies can be used, however the political causes of the observed inequities must be attributed and dismantled. Examples include studies that observe true racial inequities in cardiovascular outcomes but attribute them to biological differences.  *DISCARD:*  Race is used as a biologic construct with significant threats to internal and/or external validity. The results should be challenged, and the conclusions not applied. This includes studies that suggest differential diagnostic tools, treatment algorithms or interventions based on biologic definitions of race, for example studies used to purport racial differences in glomerular filtration rate or expected lung function. |
