# Supplementary material for: From Race to Racism: Teaching a Tool to Critically Appraise the Use of Race in Medical Research
Source: MedEdPORTAL. 2022 Jan 24;18:11210. doi: 10.15766/mep_2374-8265.11210 (PMC8784584; doi:10.15766/mep_2374-8265.11210)
Supplement: Supplementary file 1 — CARMeL Tool.docxCARMeL Workshop.pptxFacilitator Guide.docxParticipant Guide.docxUME Postsession Assessment.docxGME Pre- and Postsession Survey.docx [file mep_2374-8265.11210-s001.zip › B. CARMeL Workshop.pptx]

## Slide 1
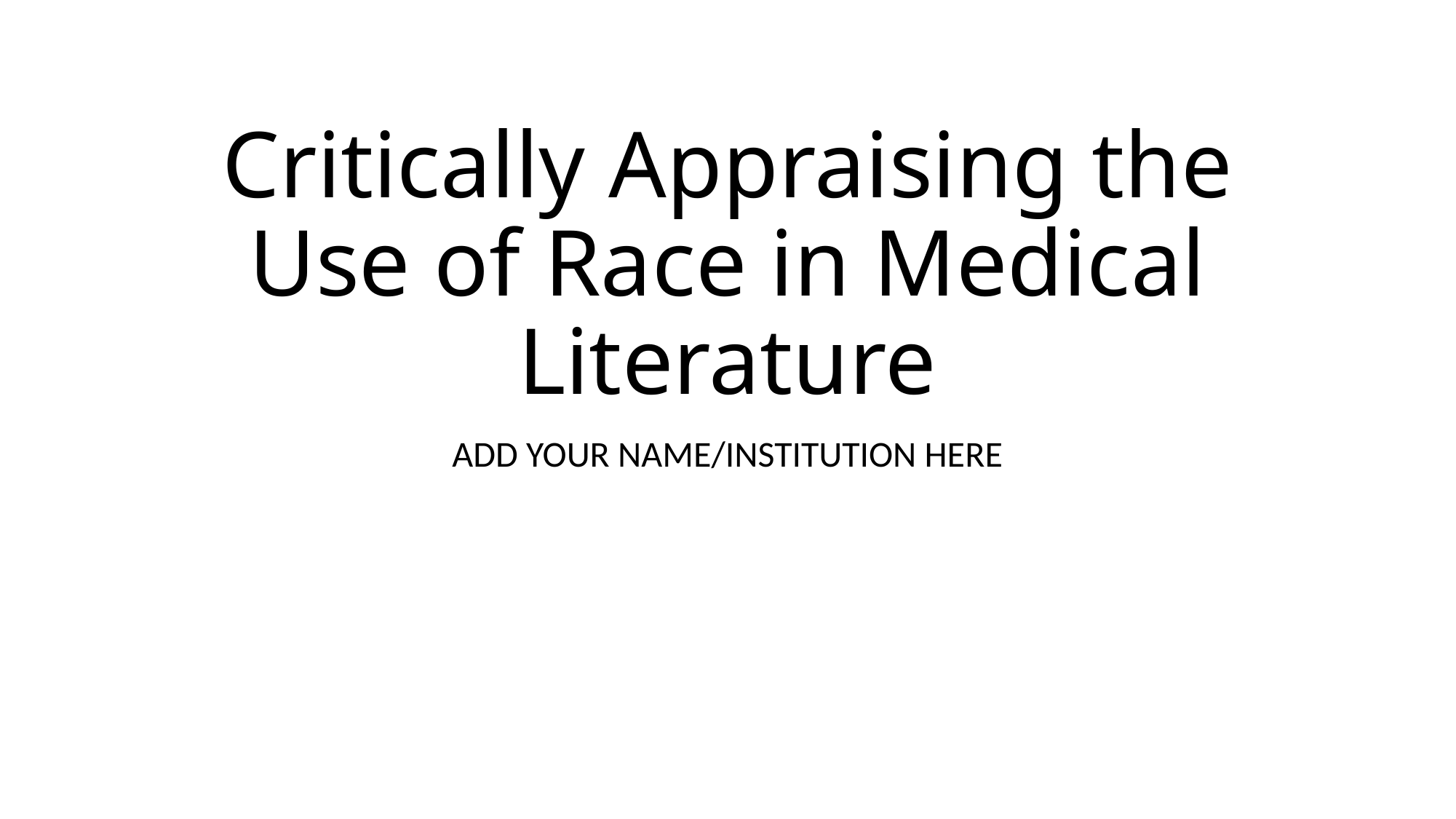

# Critically Appraising the Use of Race in Medical Literature
ADD YOUR NAME/INSTITUTION HERE

## Slide 2
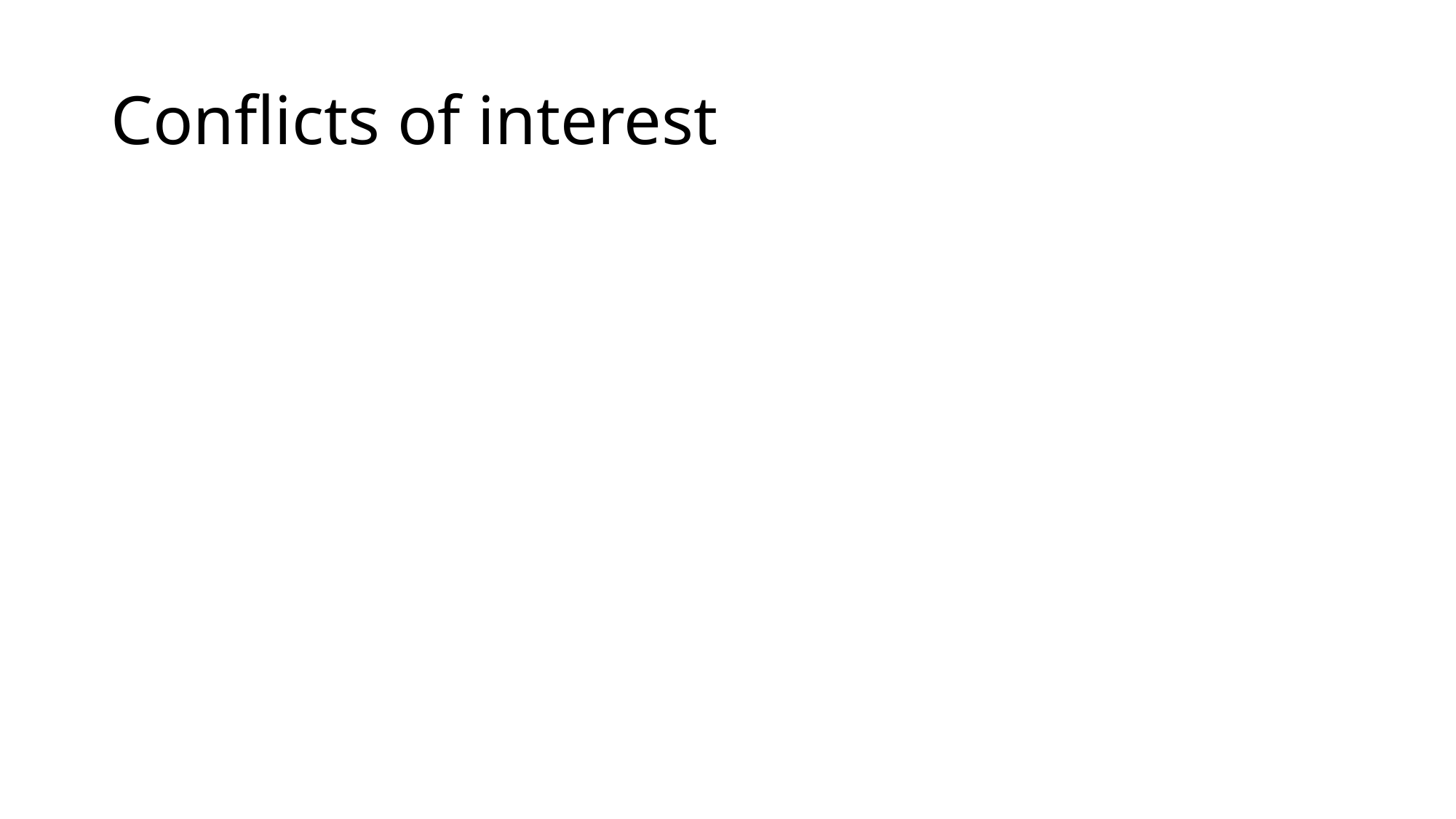

# Conflicts of interest

## Slide 3
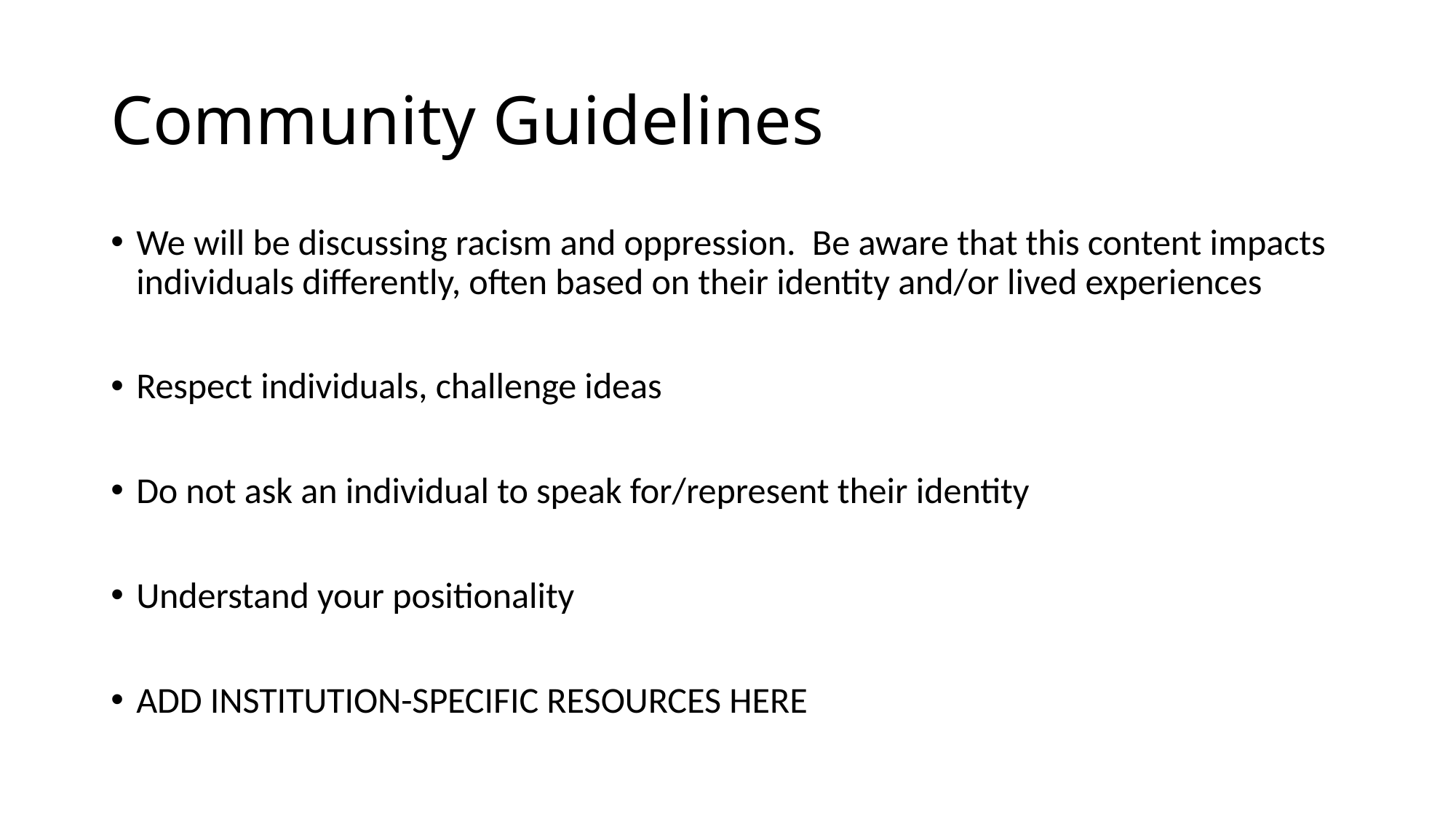

# Community Guidelines
We will be discussing racism and oppression. Be aware that this content impacts individuals differently, often based on their identity and/or lived experiences
Respect individuals, challenge ideas
Do not ask an individual to speak for/represent their identity
Understand your positionality
ADD INSTITUTION-SPECIFIC RESOURCES HERE

## Slide 4
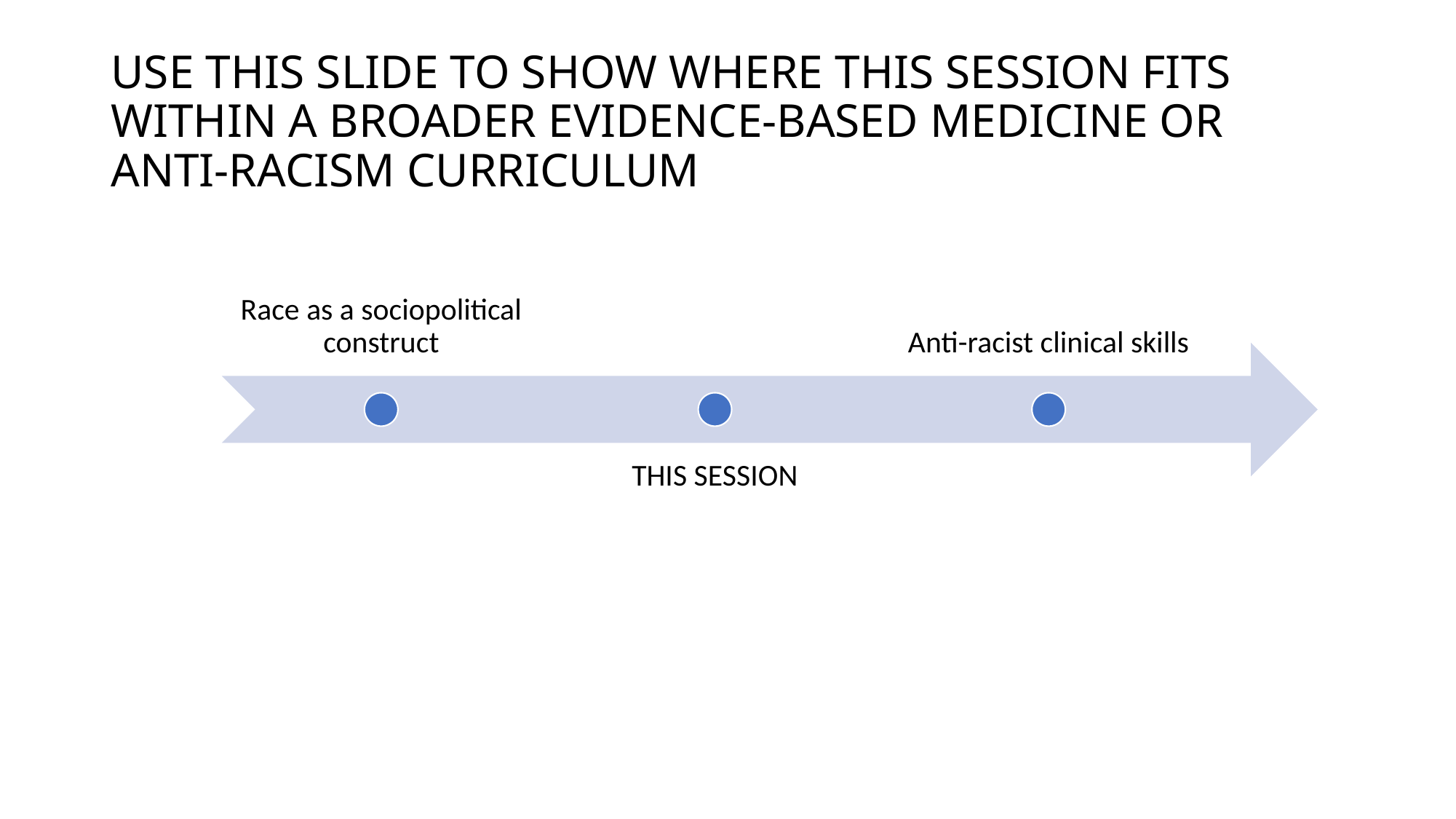

# USE THIS SLIDE TO SHOW WHERE THIS SESSION FITS WITHIN A BROADER EVIDENCE-BASED MEDICINE OR ANTI-RACISM CURRICULUM

## Slide 5
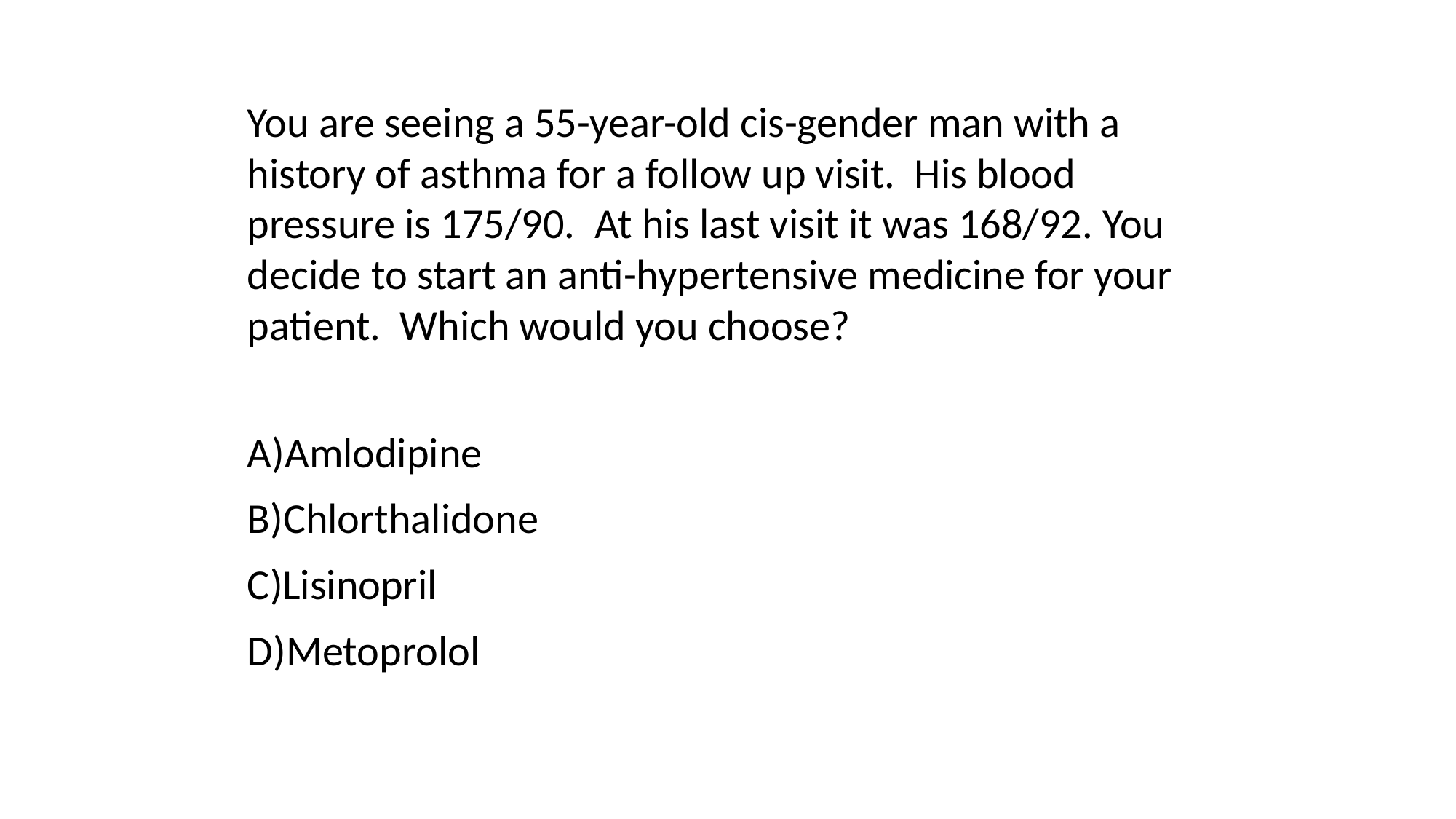

You are seeing a 55-year-old cis-gender man with a history of asthma for a follow up visit. His blood pressure is 175/90. At his last visit it was 168/92. You decide to start an anti-hypertensive medicine for your patient. Which would you choose?
Amlodipine
Chlorthalidone
Lisinopril
Metoprolol

## Slide 6
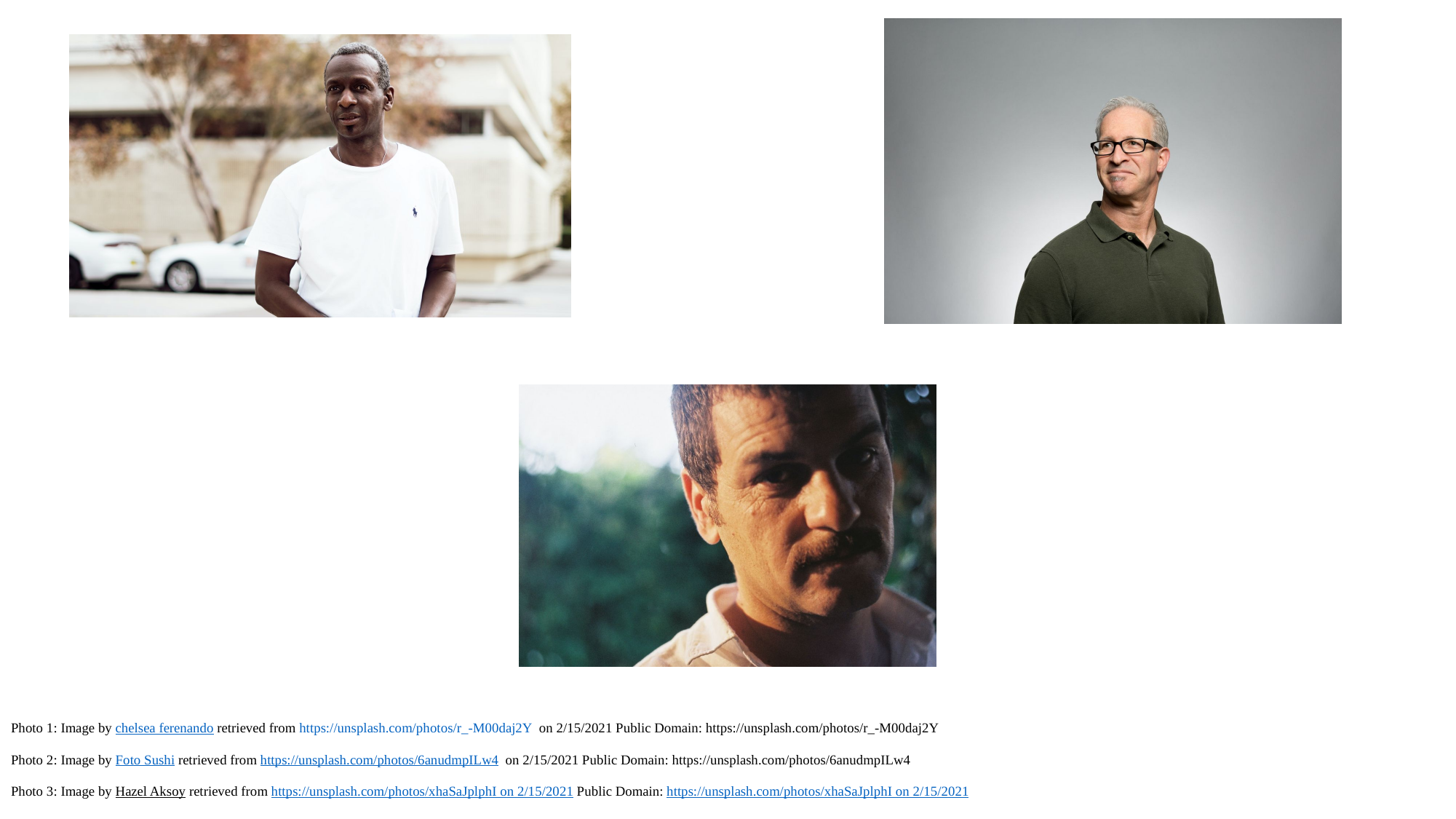

Photo 1: Image by chelsea ferenando retrieved from https://unsplash.com/photos/r_-M00daj2Y on 2/15/2021 Public Domain: https://unsplash.com/photos/r_-M00daj2Y
Photo 2: Image by Foto Sushi retrieved from https://unsplash.com/photos/6anudmpILw4 on 2/15/2021 Public Domain: https://unsplash.com/photos/6anudmpILw4
Photo 3: Image by Hazel Aksoy retrieved from https://unsplash.com/photos/xhaSaJplphI on 2/15/2021 Public Domain: https://unsplash.com/photos/xhaSaJplphI on 2/15/2021

## Slide 7
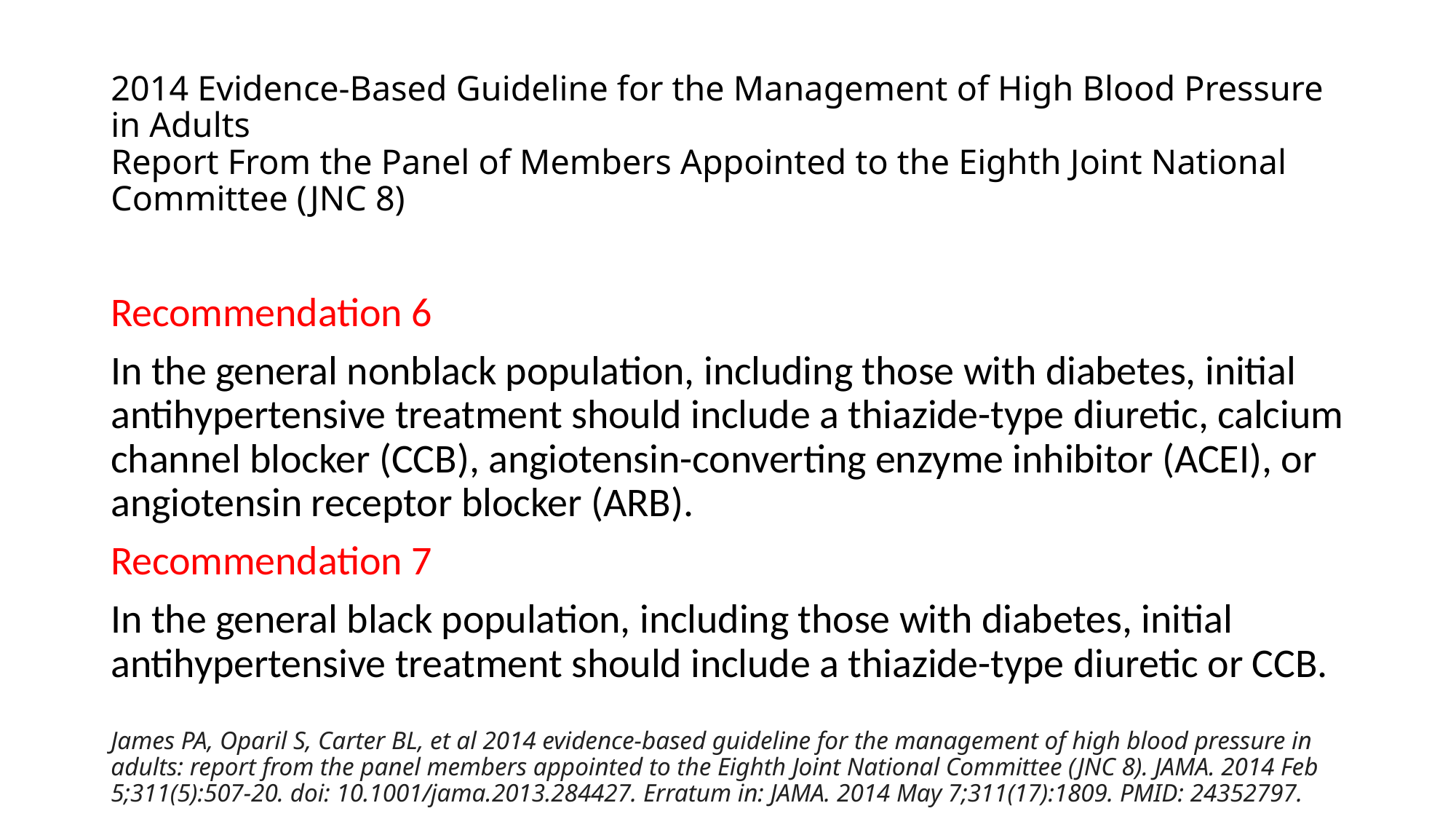

# 2014 Evidence-Based Guideline for the Management of High Blood Pressure in Adults Report From the Panel of Members Appointed to the Eighth Joint National Committee (JNC 8)
Recommendation 6
In the general nonblack population, including those with diabetes, initial antihypertensive treatment should include a thiazide-type diuretic, calcium channel blocker (CCB), angiotensin-converting enzyme inhibitor (ACEI), or angiotensin receptor blocker (ARB).
Recommendation 7
In the general black population, including those with diabetes, initial antihypertensive treatment should include a thiazide-type diuretic or CCB.
James PA, Oparil S, Carter BL, et al 2014 evidence-based guideline for the management of high blood pressure in adults: report from the panel members appointed to the Eighth Joint National Committee (JNC 8). JAMA. 2014 Feb 5;311(5):507-20. doi: 10.1001/jama.2013.284427. Erratum in: JAMA. 2014 May 7;311(17):1809. PMID: 24352797.

## Slide 8
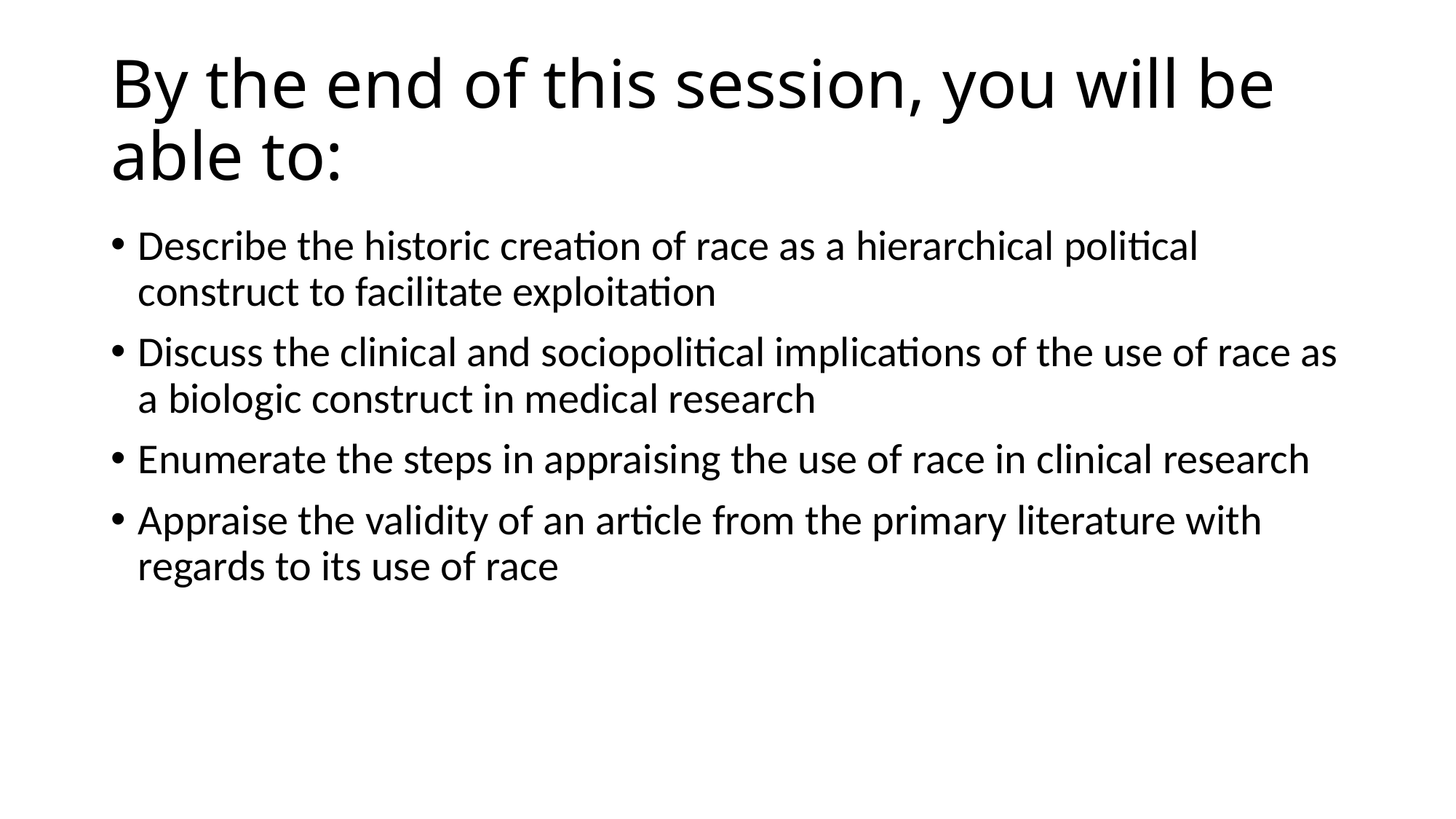

# By the end of this session, you will be able to:
Describe the historic creation of race as a hierarchical political construct to facilitate exploitation
Discuss the clinical and sociopolitical implications of the use of race as a biologic construct in medical research
Enumerate the steps in appraising the use of race in clinical research
Appraise the validity of an article from the primary literature with regards to its use of race

## Slide 9
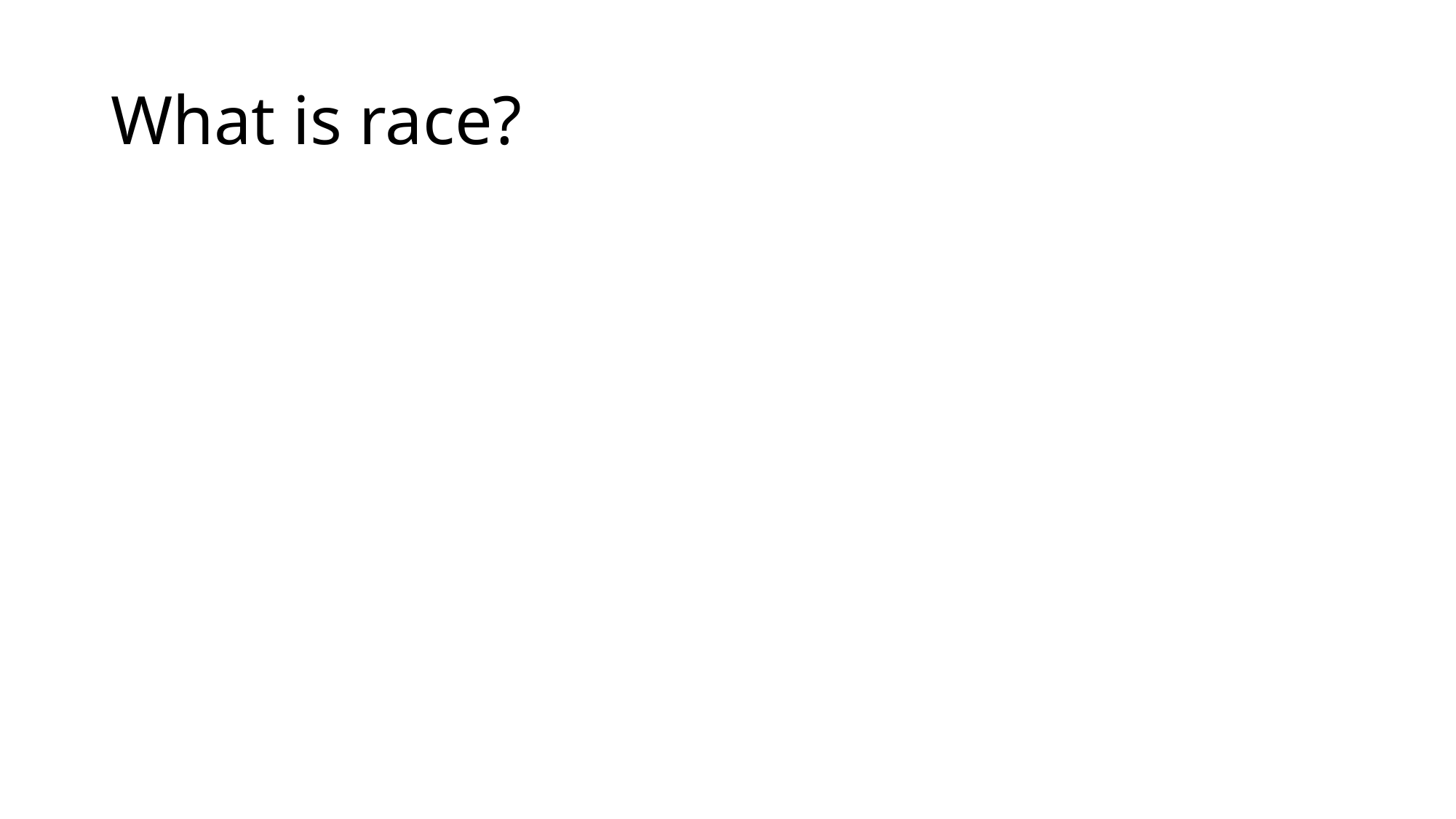

# What is race?

## Slide 10
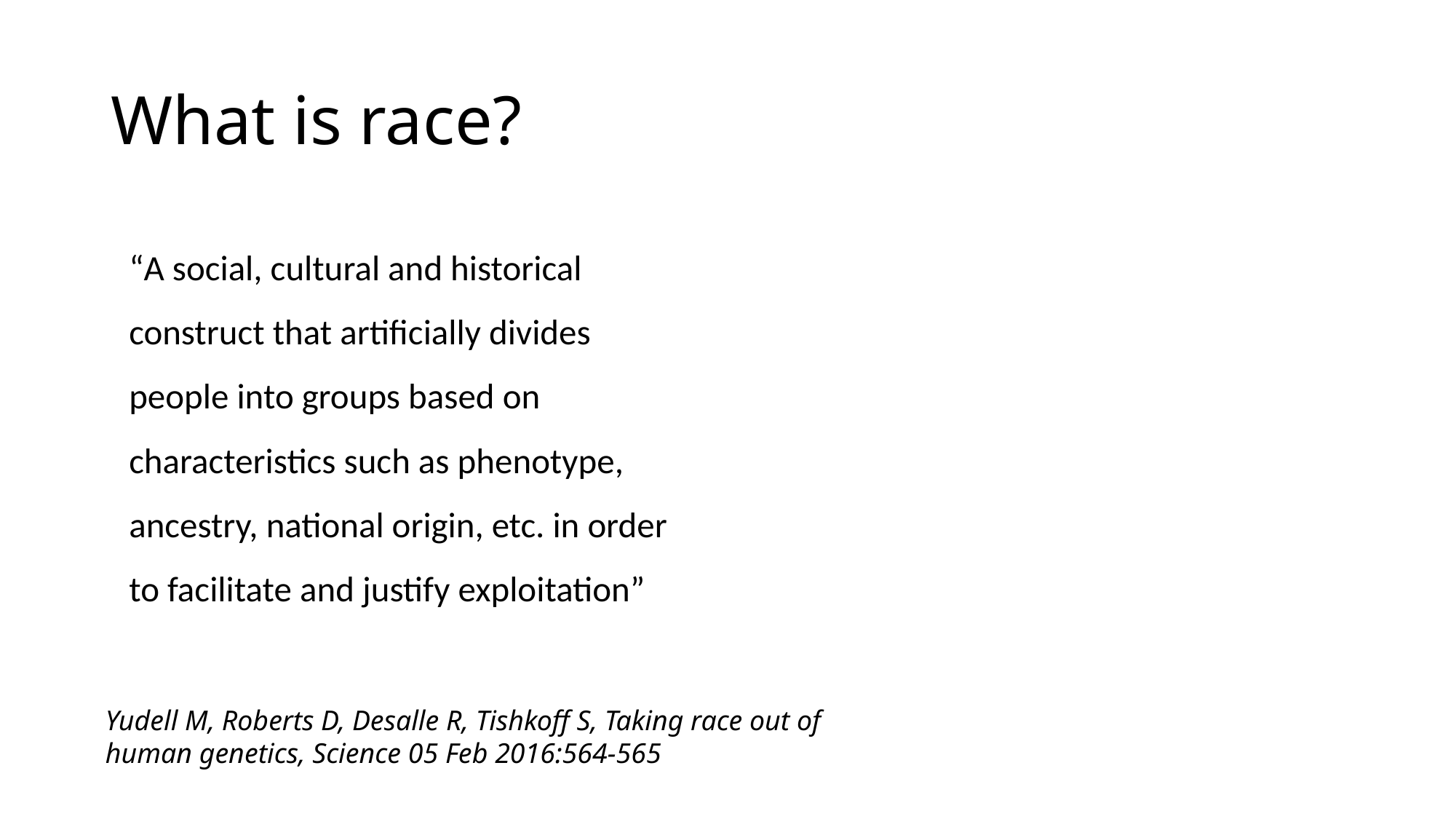

# What is race?
“A social, cultural and historical construct that artificially divides people into groups based on characteristics such as phenotype, ancestry, national origin, etc. in order to facilitate and justify exploitation”
Yudell M, Roberts D, Desalle R, Tishkoff S, Taking race out of human genetics, Science 05 Feb 2016:564-565

## Slide 11
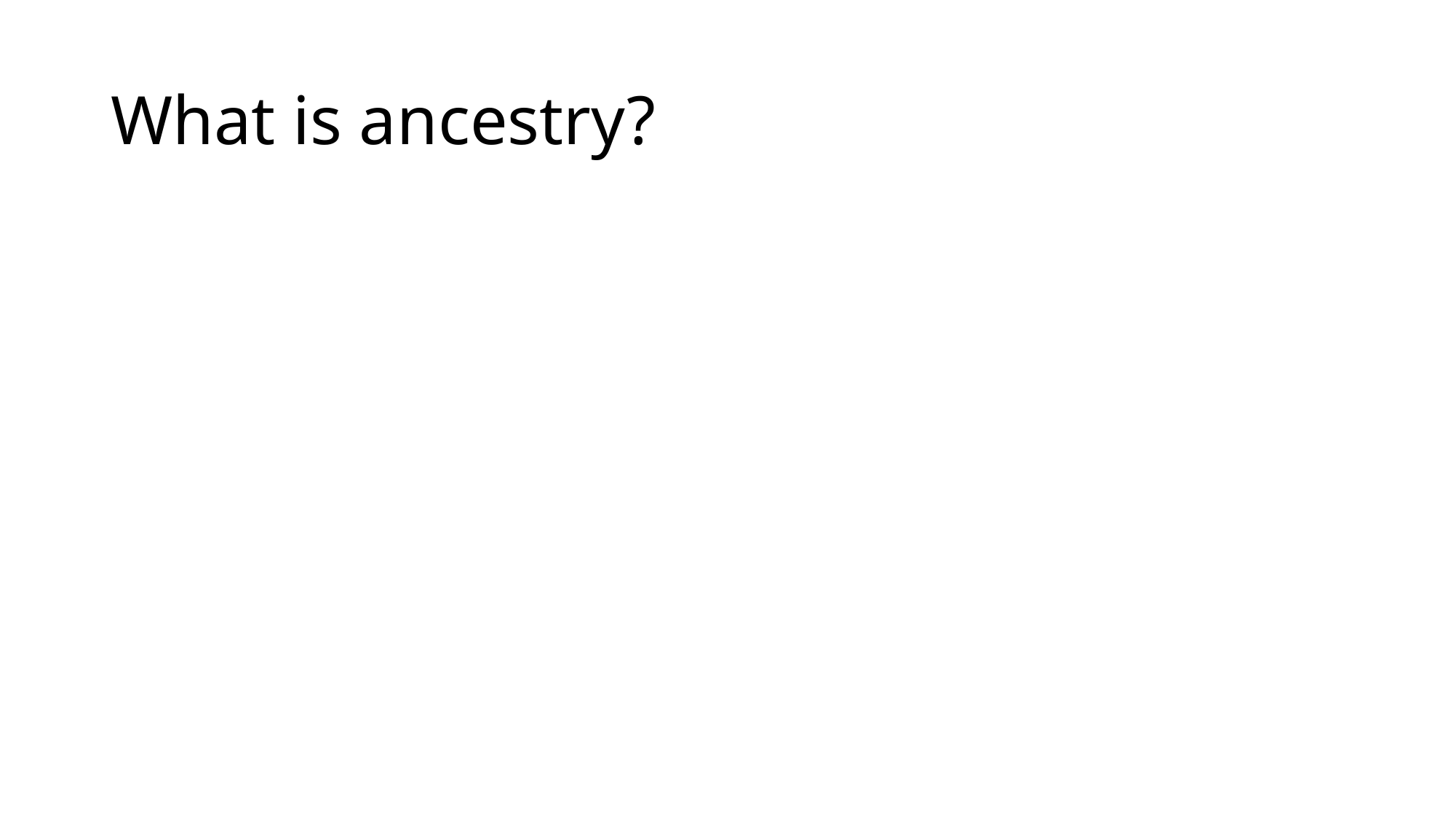

# What is ancestry?

## Slide 12
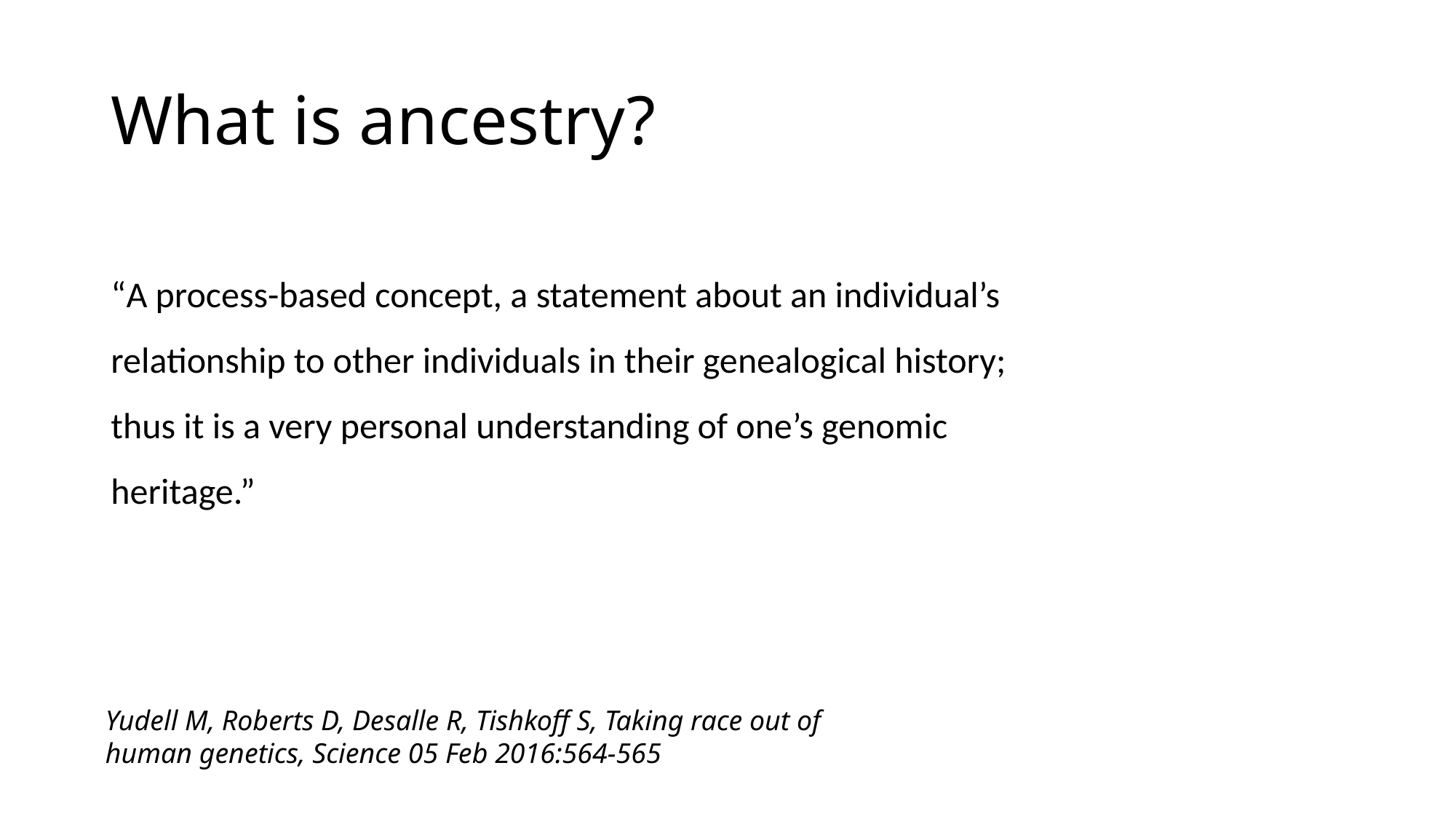

# What is ancestry?
“A process-based concept, a statement about an individual’s relationship to other individuals in their genealogical history; thus it is a very personal understanding of one’s genomic heritage.”
Yudell M, Roberts D, Desalle R, Tishkoff S, Taking race out of human genetics, Science 05 Feb 2016:564-565

## Slide 13
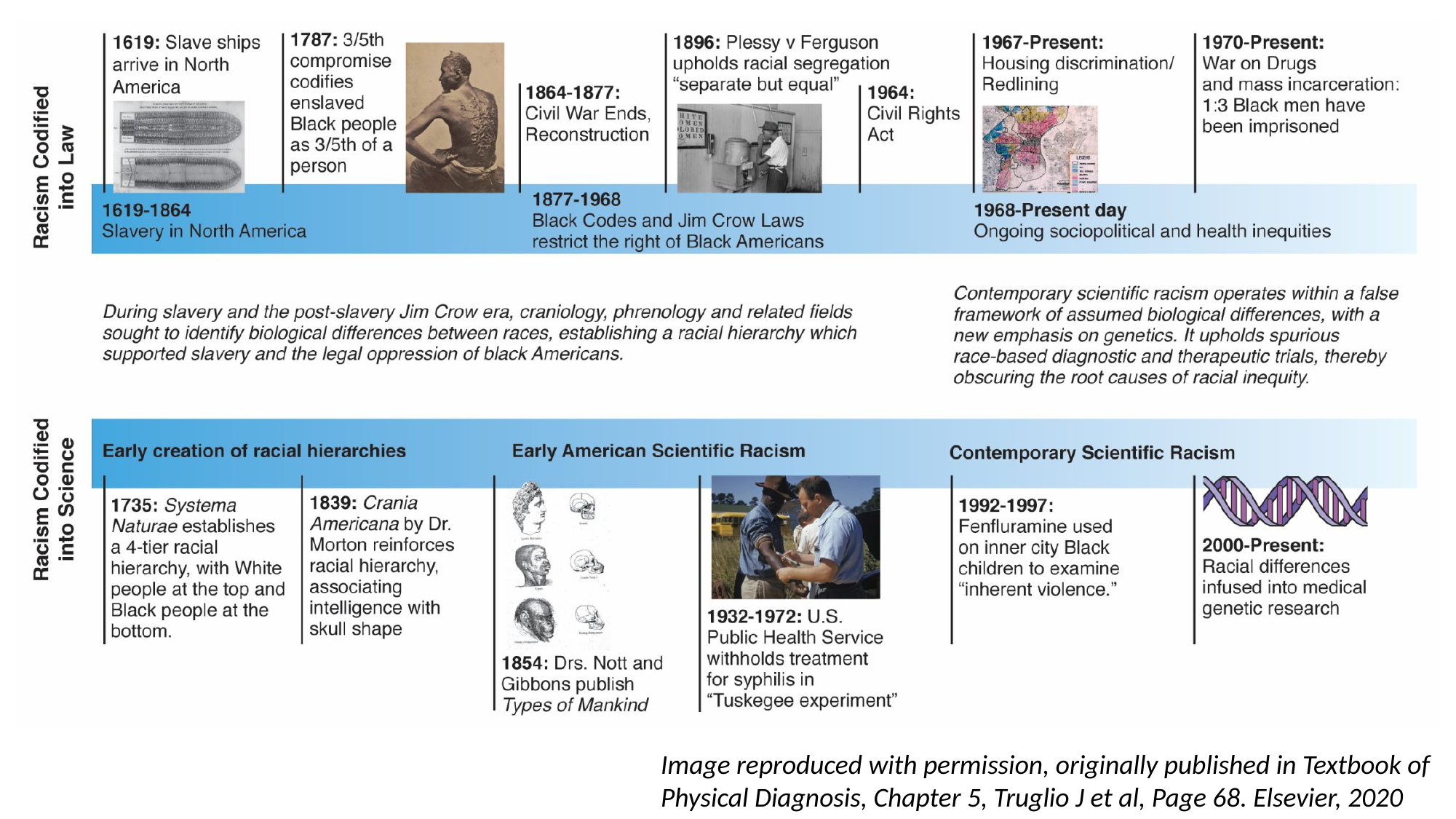

Image reproduced with permission, originally published in Textbook of Physical Diagnosis, Chapter 5, Truglio J et al, Page 68. Elsevier, 2020

## Slide 14
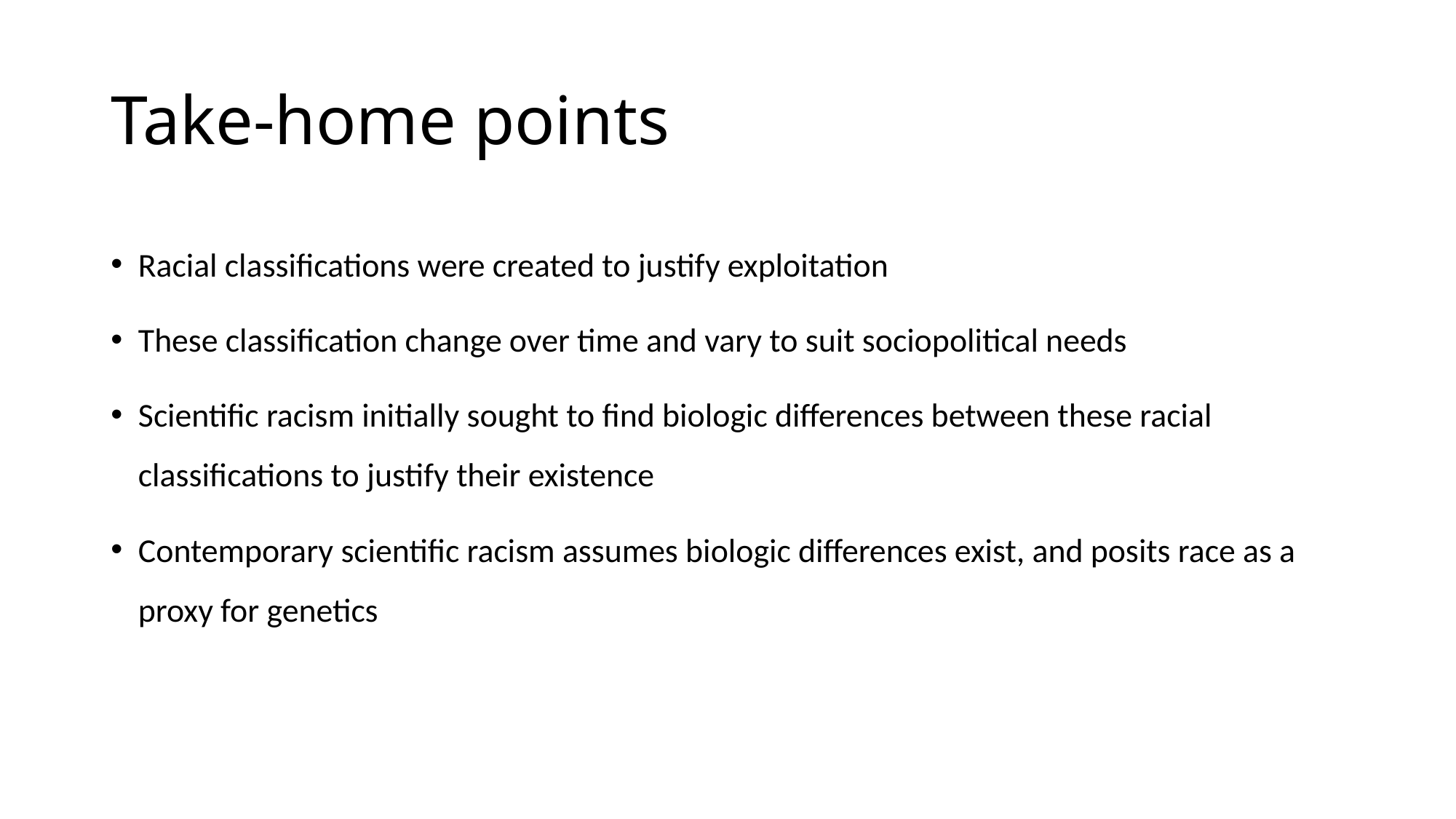

# Take-home points
Racial classifications were created to justify exploitation
These classification change over time and vary to suit sociopolitical needs
Scientific racism initially sought to find biologic differences between these racial classifications to justify their existence
Contemporary scientific racism assumes biologic differences exist, and posits race as a proxy for genetics

## Slide 15
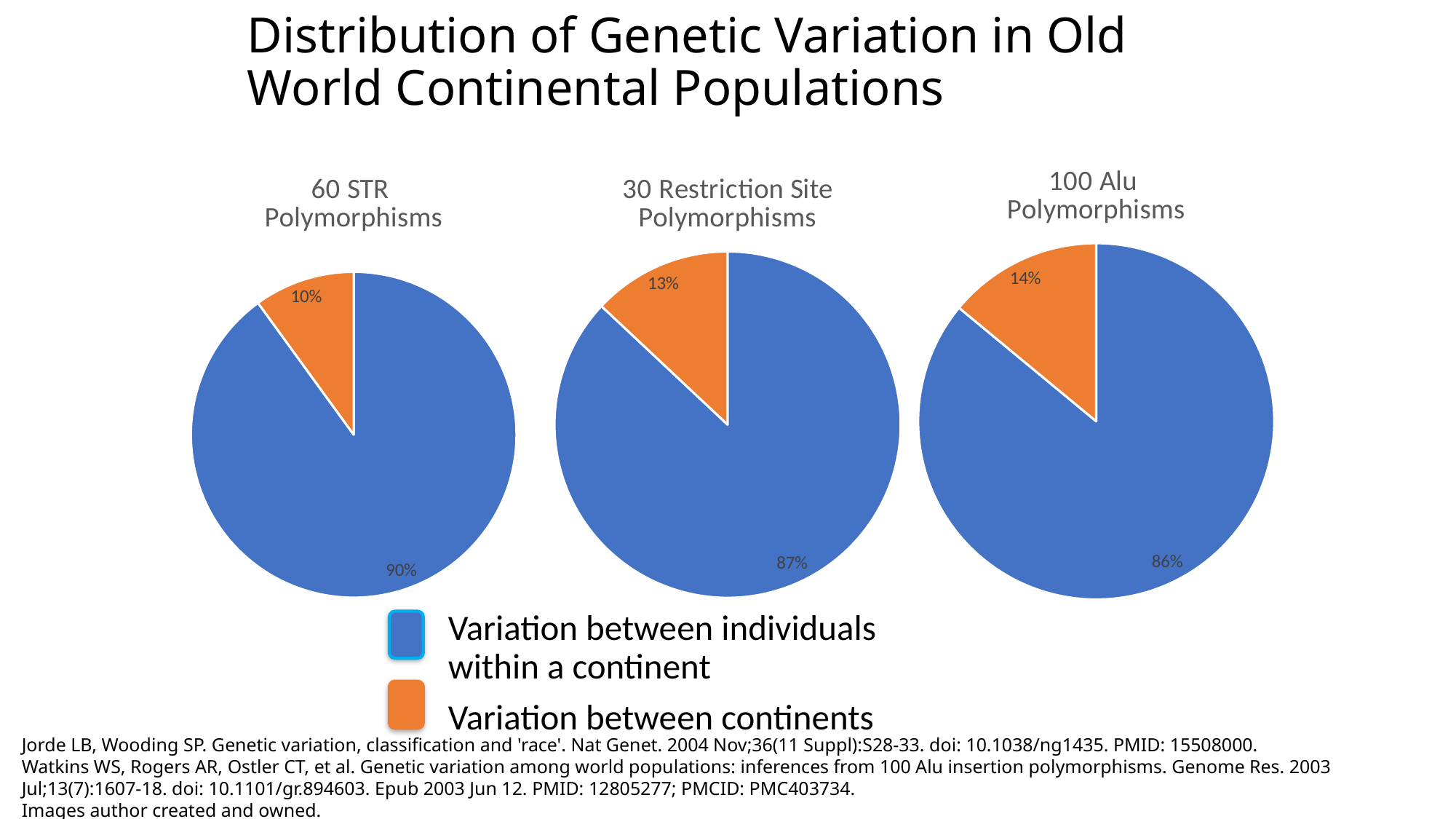

# Distribution of Genetic Variation in Old World Continental Populations
### Chart: 100 Alu
Polymorphisms
| Category | 60 STR |
|---|---|
| Between individuals within continents | 0.86 |
| Between continents | 0.14 |
### Chart: 60 STR
Polymorphisms
| Category | 60 STR Polymorphisms |
|---|---|
| Between individuals within continents | 0.9 |
| Between continents | 0.1 |
### Chart:
| Category | 30 Restriction Site Polymorphisms |
|---|---|
| Between individuals within continents | 0.87 |
| Between continents | 0.13 |Variation between individuals within a continent
Variation between continents
Jorde LB, Wooding SP. Genetic variation, classification and 'race'. Nat Genet. 2004 Nov;36(11 Suppl):S28-33. doi: 10.1038/ng1435. PMID: 15508000.
Watkins WS, Rogers AR, Ostler CT, et al. Genetic variation among world populations: inferences from 100 Alu insertion polymorphisms. Genome Res. 2003 Jul;13(7):1607-18. doi: 10.1101/gr.894603. Epub 2003 Jun 12. PMID: 12805277; PMCID: PMC403734.
Images author created and owned.

## Slide 16
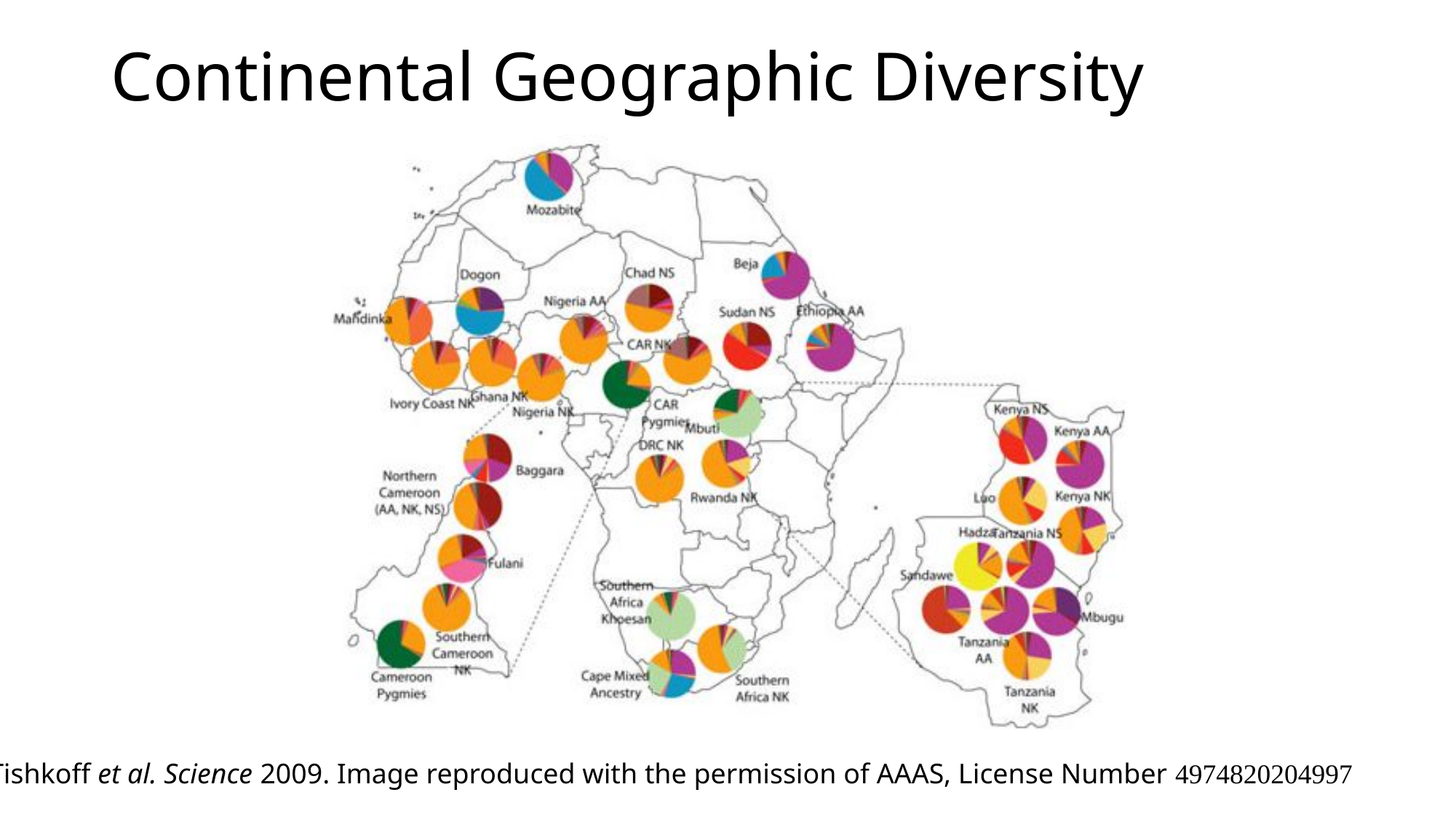

# Continental Geographic Diversity
Tishkoff et al. Science 2009. Image reproduced with the permission of AAAS, License Number 4974820204997

## Slide 17
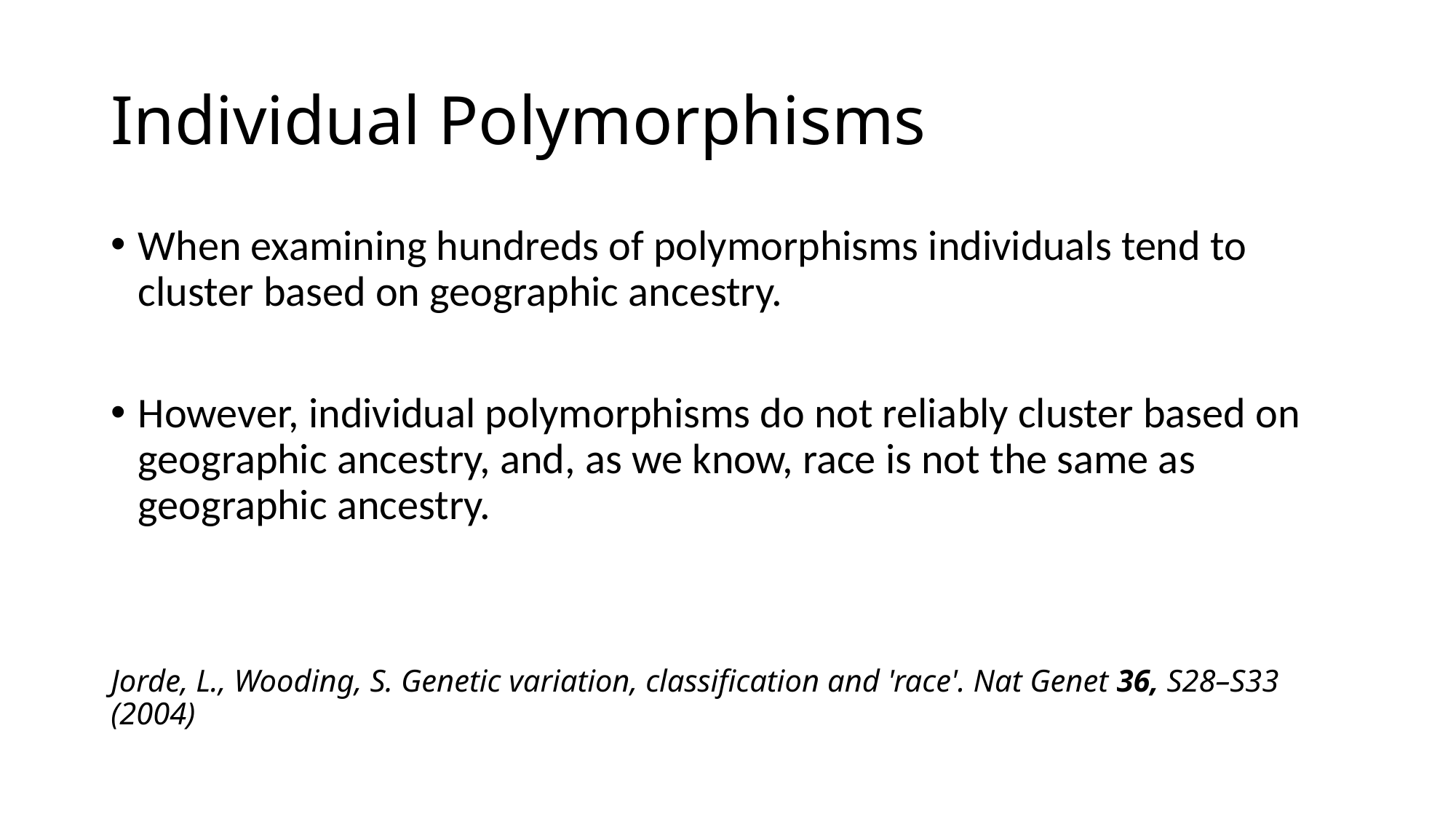

# Individual Polymorphisms
When examining hundreds of polymorphisms individuals tend to cluster based on geographic ancestry.
However, individual polymorphisms do not reliably cluster based on geographic ancestry, and, as we know, race is not the same as geographic ancestry.
Jorde, L., Wooding, S. Genetic variation, classification and 'race'. Nat Genet 36, S28–S33 (2004)

## Slide 18
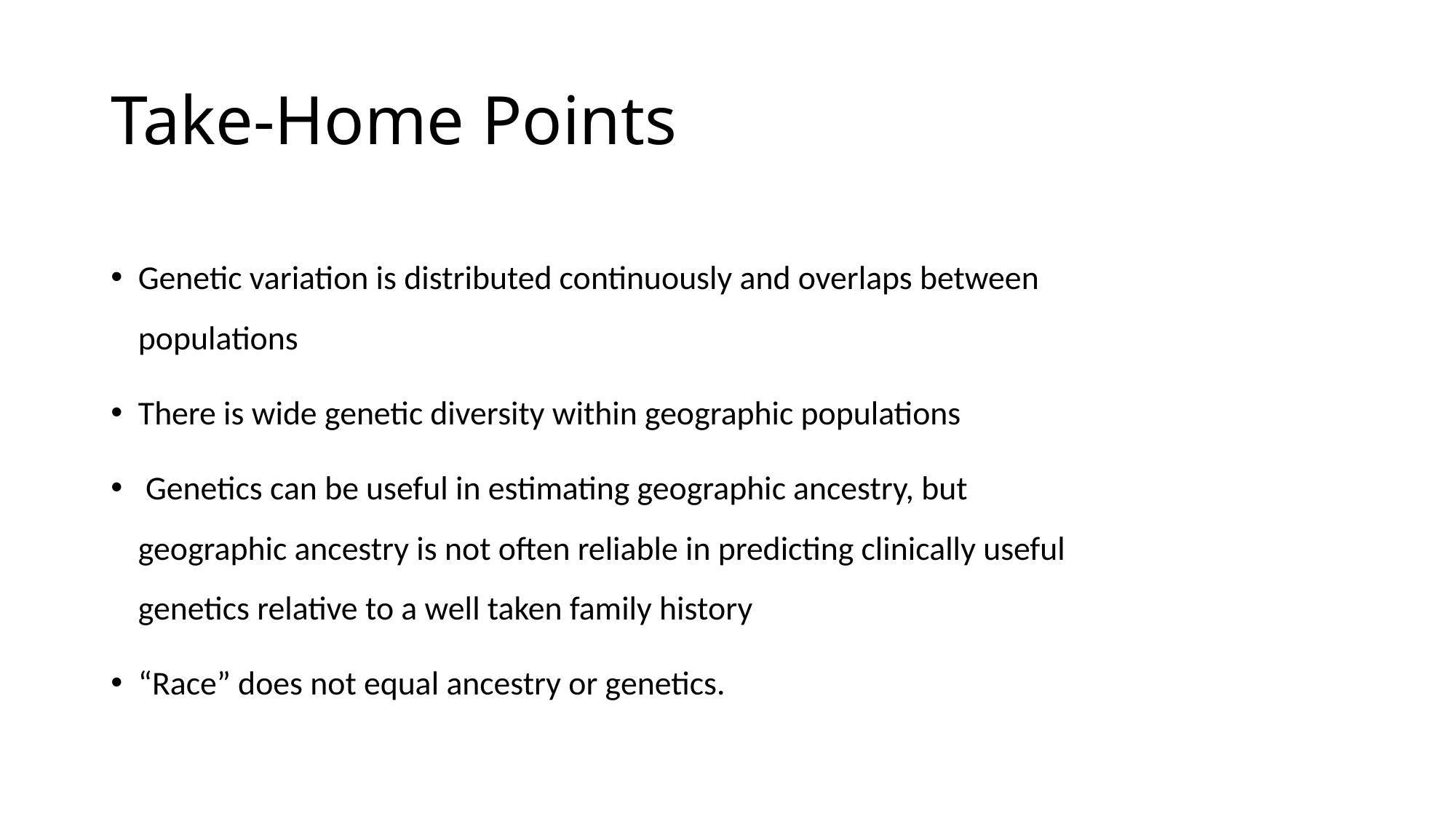

# Take-Home Points
Genetic variation is distributed continuously and overlaps between populations
There is wide genetic diversity within geographic populations
 Genetics can be useful in estimating geographic ancestry, but geographic ancestry is not often reliable in predicting clinically useful genetics relative to a well taken family history
“Race” does not equal ancestry or genetics.

## Slide 19
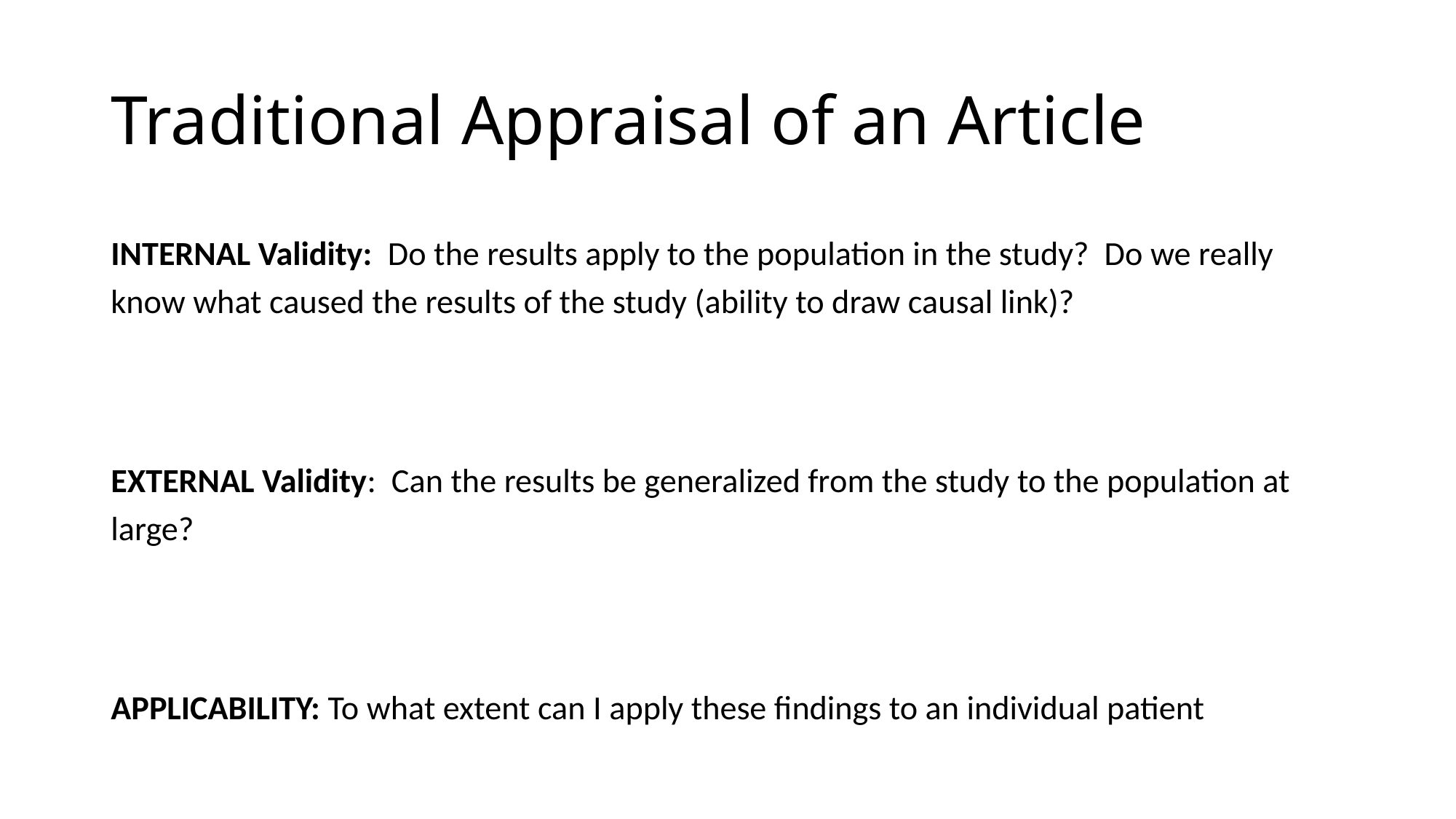

# Traditional Appraisal of an Article
INTERNAL Validity: Do the results apply to the population in the study? Do we really know what caused the results of the study (ability to draw causal link)?
EXTERNAL Validity: Can the results be generalized from the study to the population at large?
APPLICABILITY: To what extent can I apply these findings to an individual patient

## Slide 20
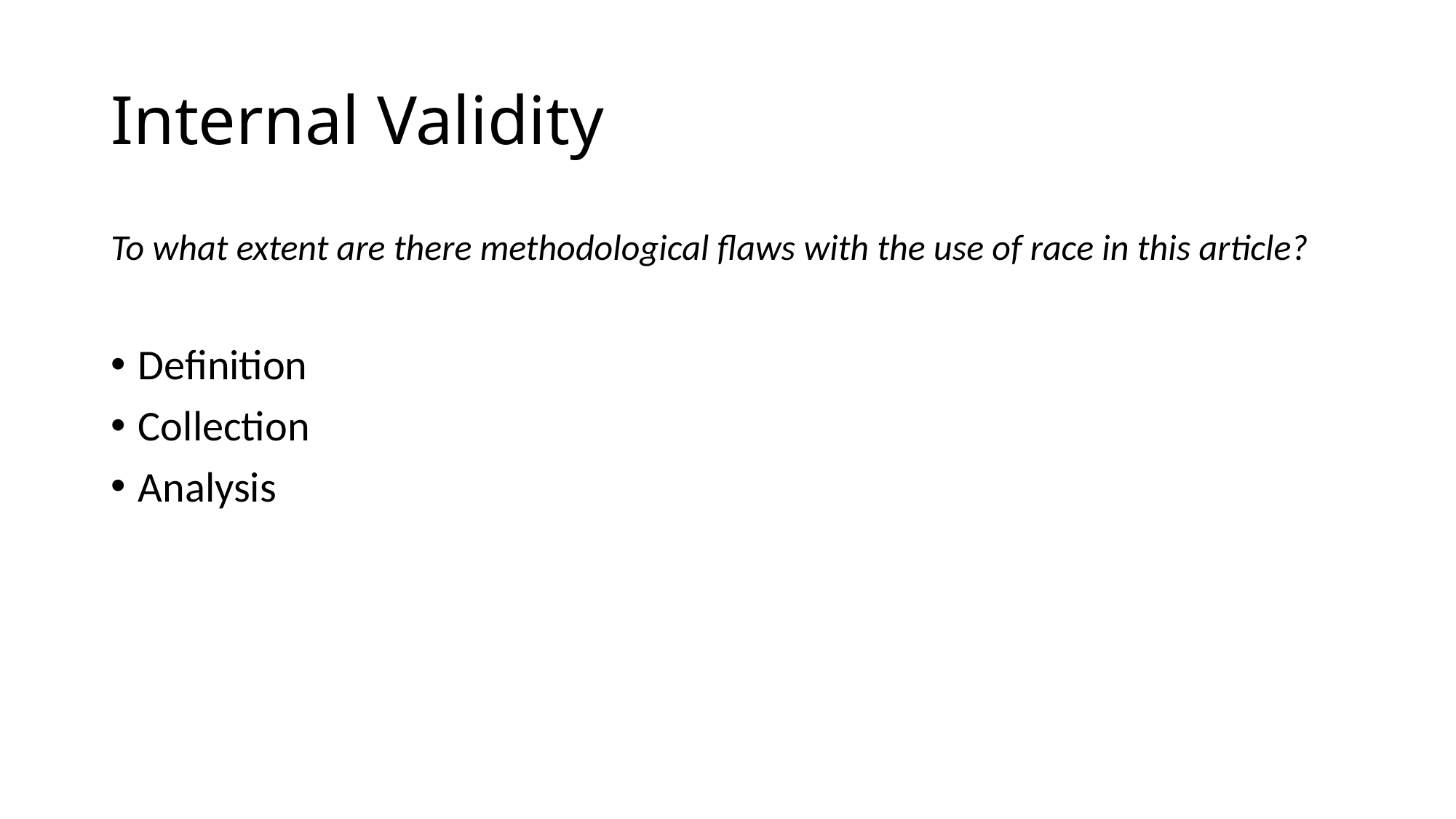

# Internal Validity
To what extent are there methodological flaws with the use of race in this article?
Definition
Collection
Analysis

## Slide 21
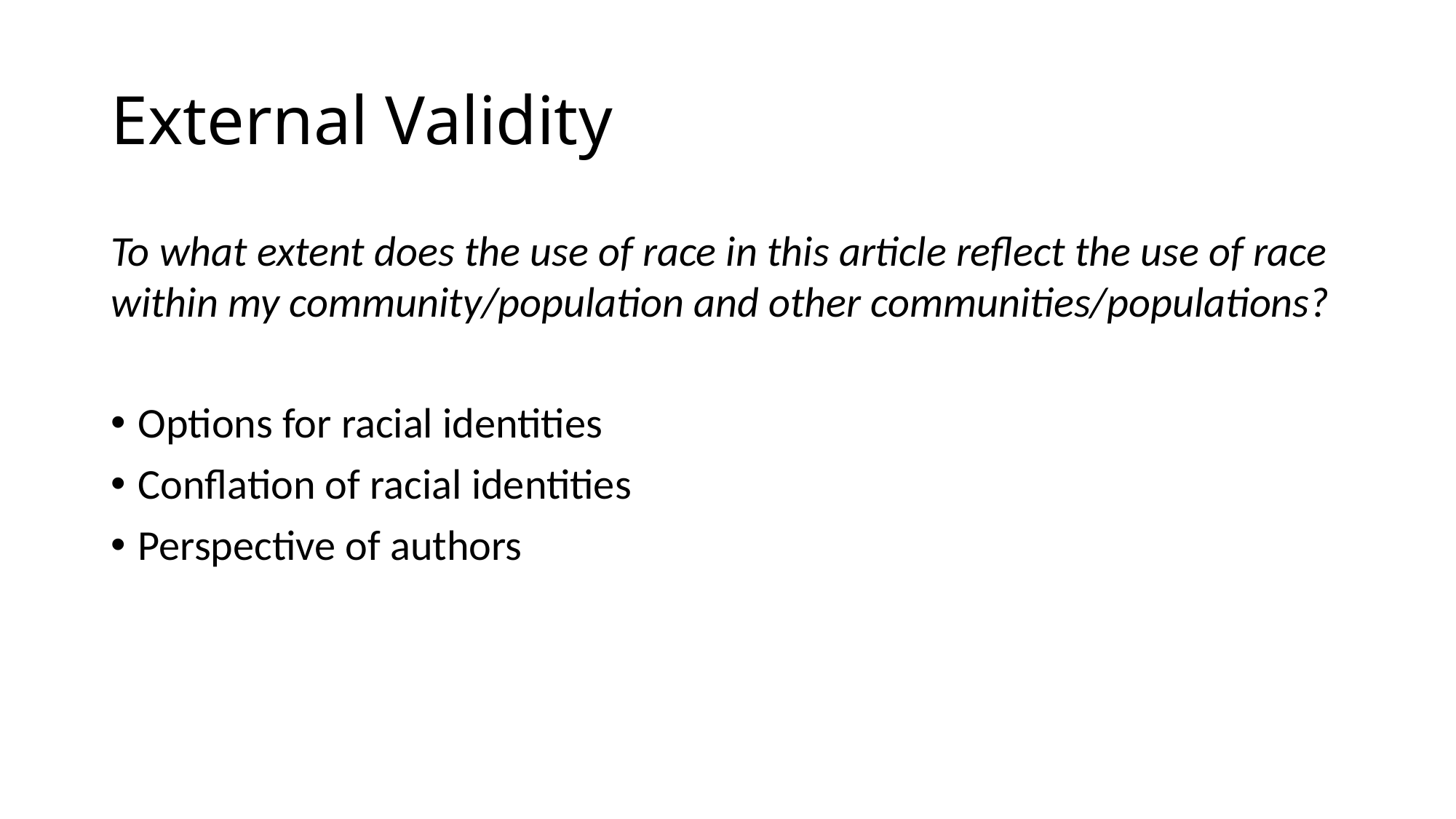

# External Validity
To what extent does the use of race in this article reflect the use of race within my community/population and other communities/populations?
Options for racial identities
Conflation of racial identities
Perspective of authors

## Slide 22
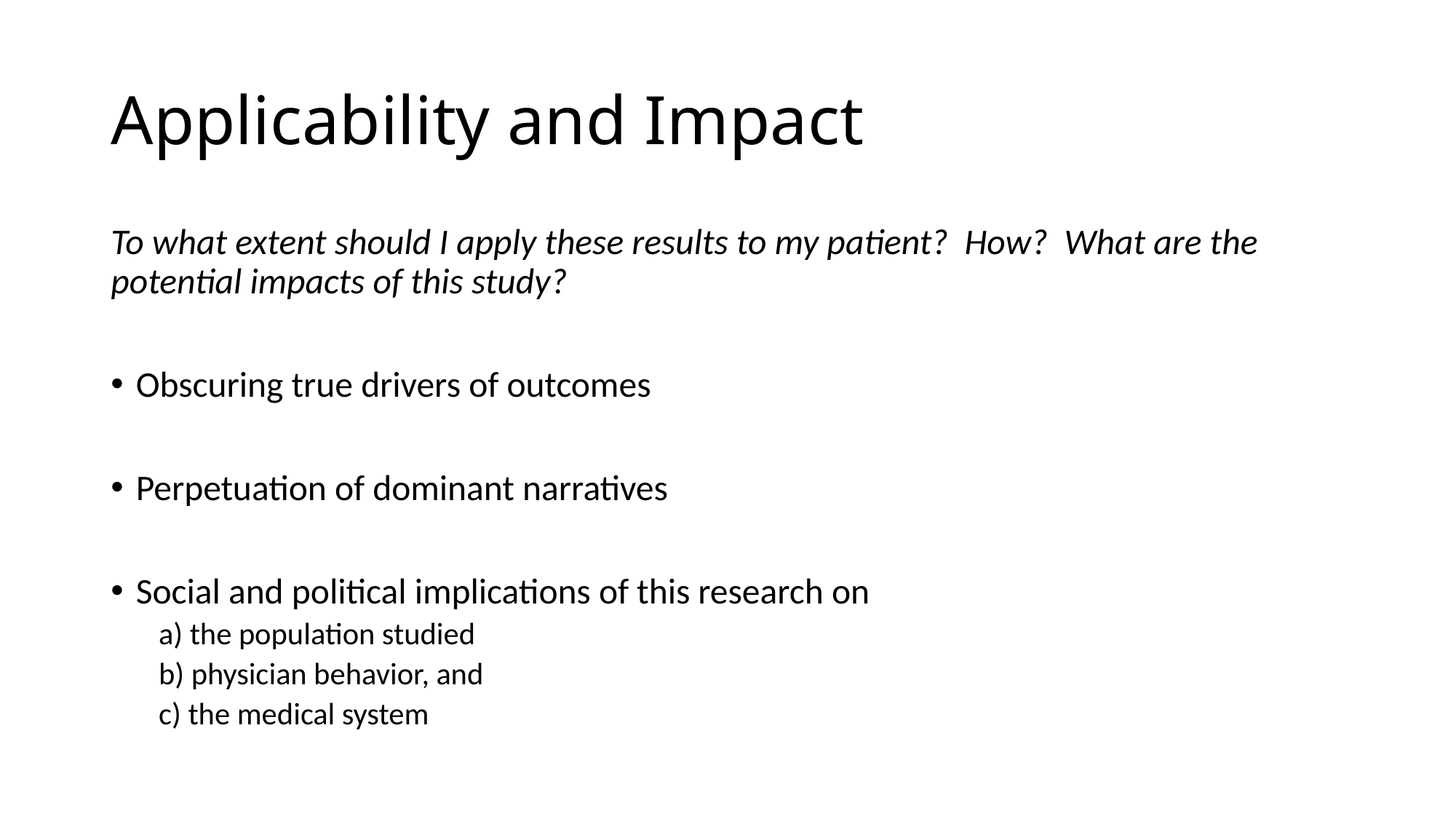

# Applicability and Impact
To what extent should I apply these results to my patient? How? What are the potential impacts of this study?
Obscuring true drivers of outcomes
Perpetuation of dominant narratives
Social and political implications of this research on
a) the population studied
b) physician behavior, and
c) the medical system

## Slide 23
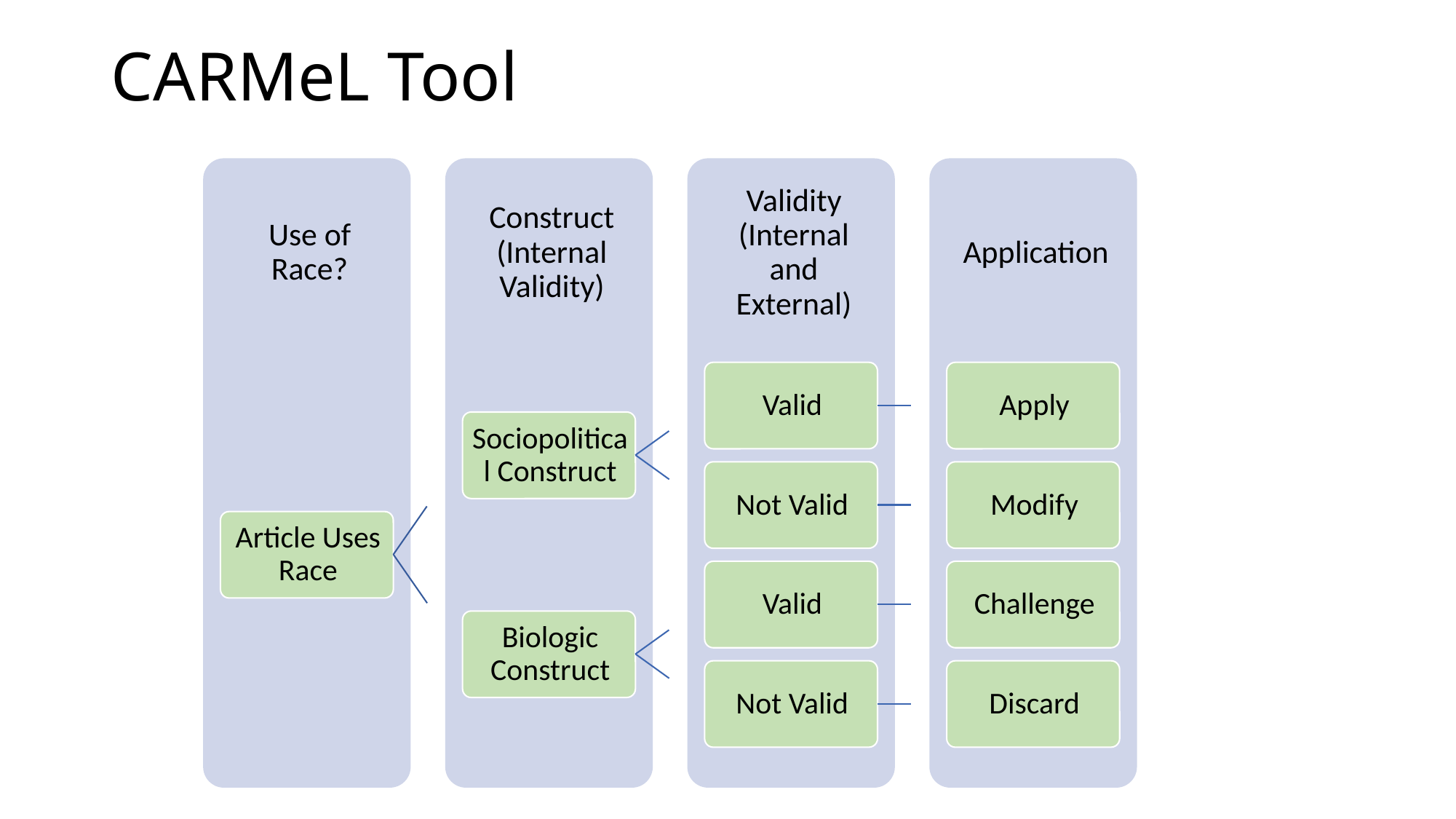

# CARMeL Tool

## Slide 24
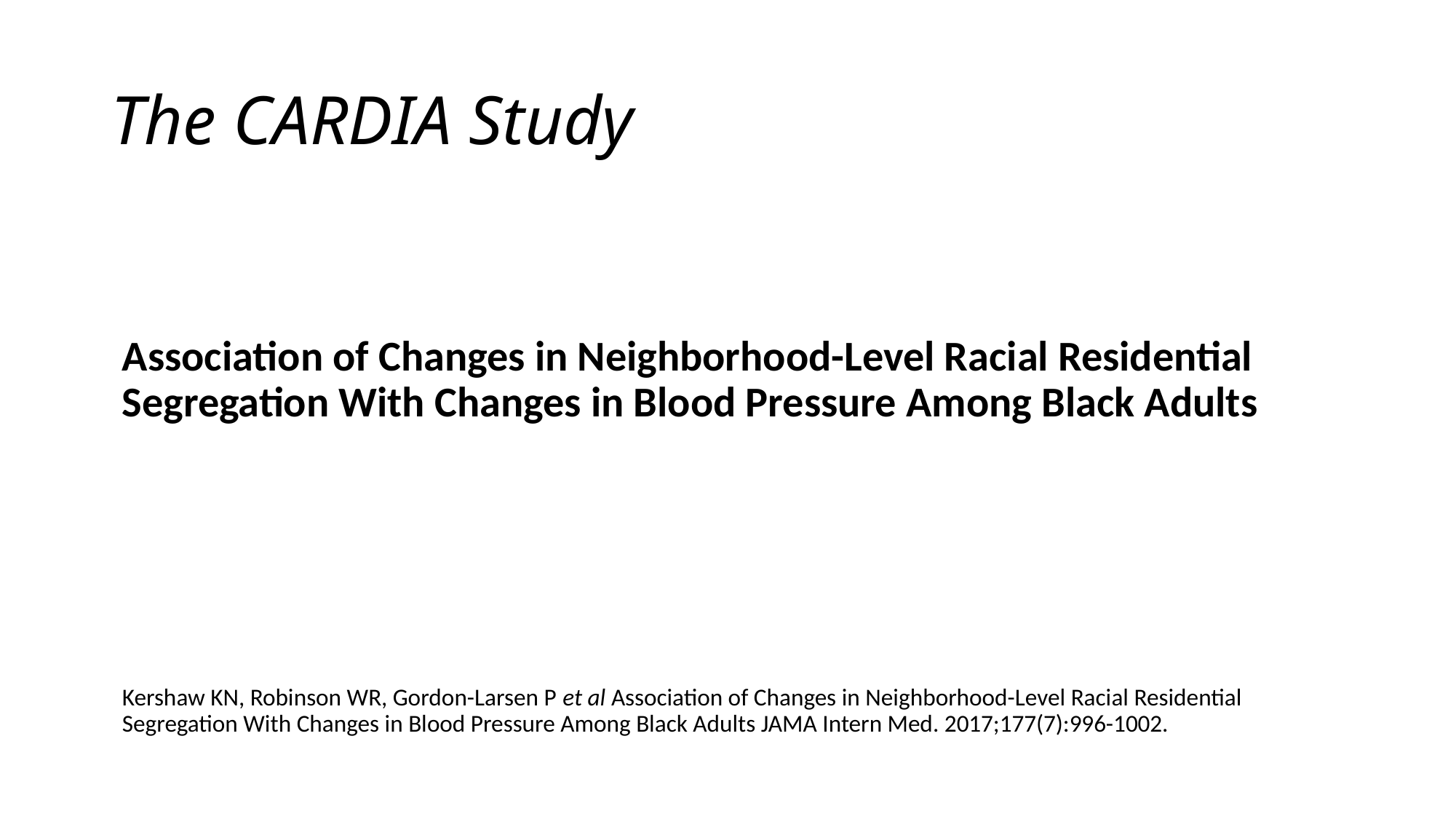

# The CARDIA Study
Association of Changes in Neighborhood-Level Racial Residential Segregation With Changes in Blood Pressure Among Black Adults
Kershaw KN, Robinson WR, Gordon-Larsen P et al Association of Changes in Neighborhood-Level Racial Residential Segregation With Changes in Blood Pressure Among Black Adults JAMA Intern Med. 2017;177(7):996-1002.

## Slide 25
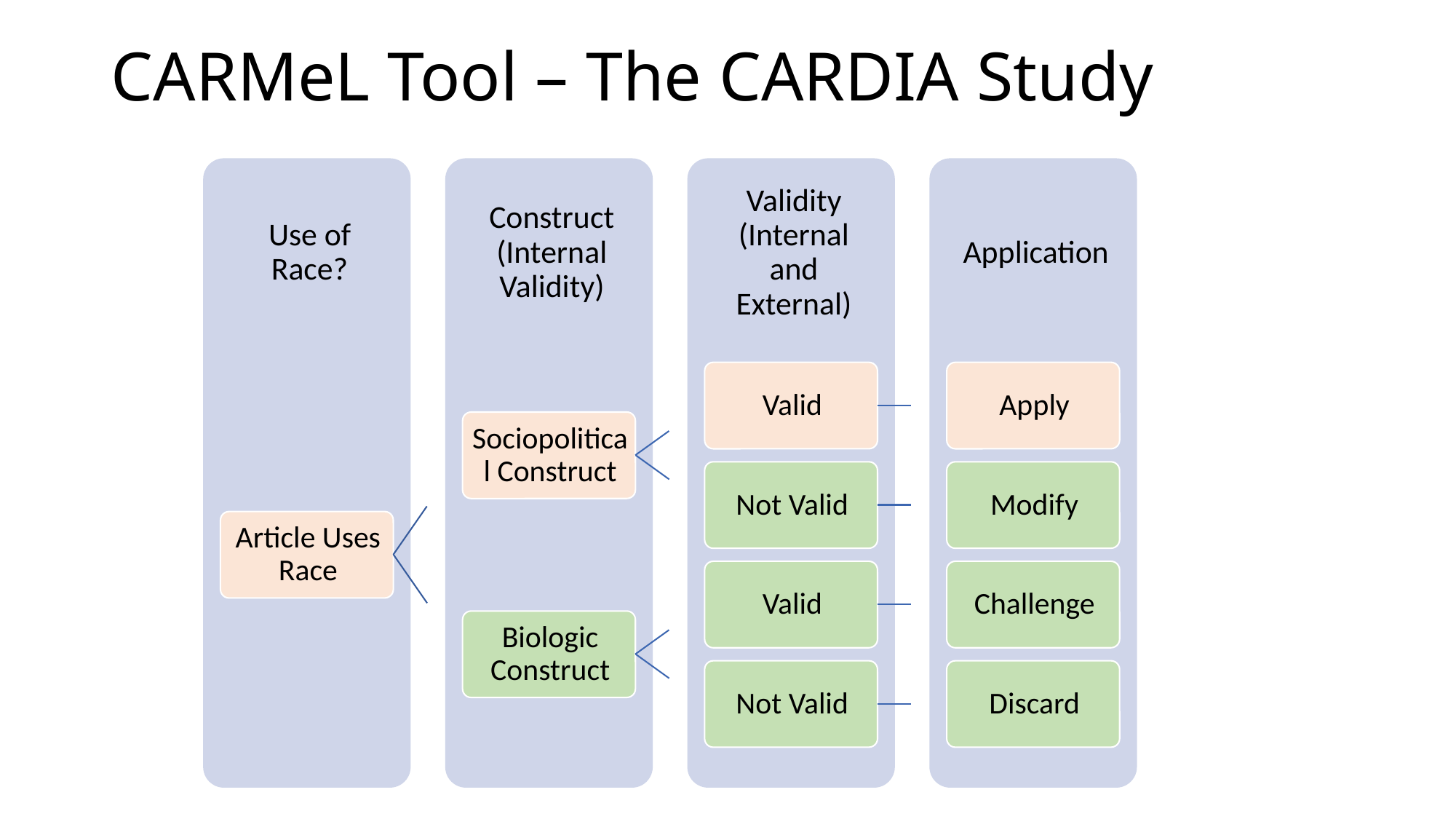

# CARMeL Tool – The CARDIA Study

## Slide 26
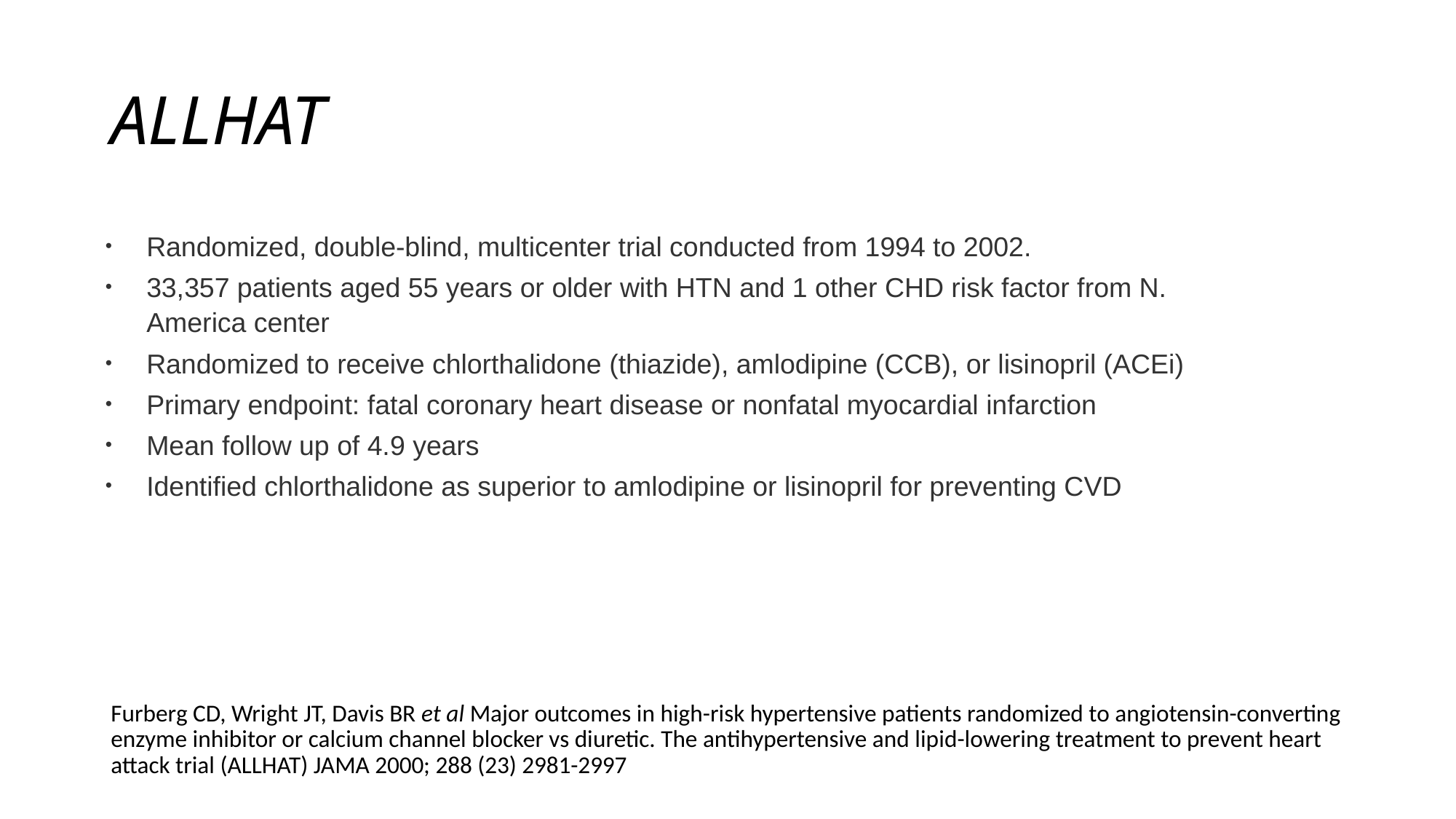

# ALLHAT
Randomized, double-blind, multicenter trial conducted from 1994 to 2002.
33,357 patients aged 55 years or older with HTN and 1 other CHD risk factor from N. America center
Randomized to receive chlorthalidone (thiazide), amlodipine (CCB), or lisinopril (ACEi)
Primary endpoint: fatal coronary heart disease or nonfatal myocardial infarction
Mean follow up of 4.9 years
Identified chlorthalidone as superior to amlodipine or lisinopril for preventing CVD
Furberg CD, Wright JT, Davis BR et al Major outcomes in high-risk hypertensive patients randomized to angiotensin-converting enzyme inhibitor or calcium channel blocker vs diuretic. The antihypertensive and lipid-lowering treatment to prevent heart attack trial (ALLHAT) JAMA 2000; 288 (23) 2981-2997

## Slide 27
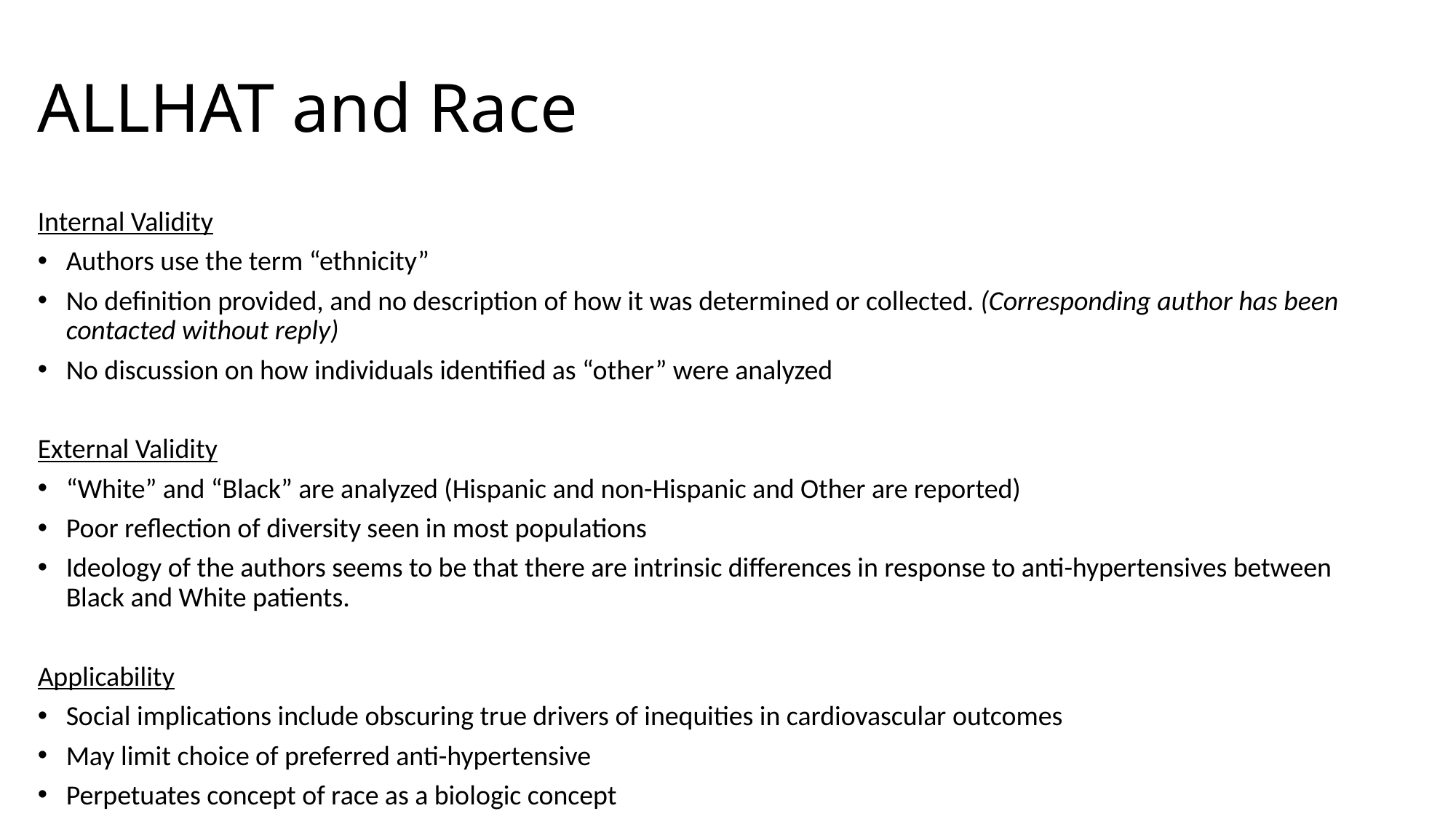

# ALLHAT and Race
Internal Validity
Authors use the term “ethnicity”
No definition provided, and no description of how it was determined or collected. (Corresponding author has been contacted without reply)
No discussion on how individuals identified as “other” were analyzed
External Validity
“White” and “Black” are analyzed (Hispanic and non-Hispanic and Other are reported)
Poor reflection of diversity seen in most populations
Ideology of the authors seems to be that there are intrinsic differences in response to anti-hypertensives between Black and White patients.
Applicability
Social implications include obscuring true drivers of inequities in cardiovascular outcomes
May limit choice of preferred anti-hypertensive
Perpetuates concept of race as a biologic concept

## Slide 28
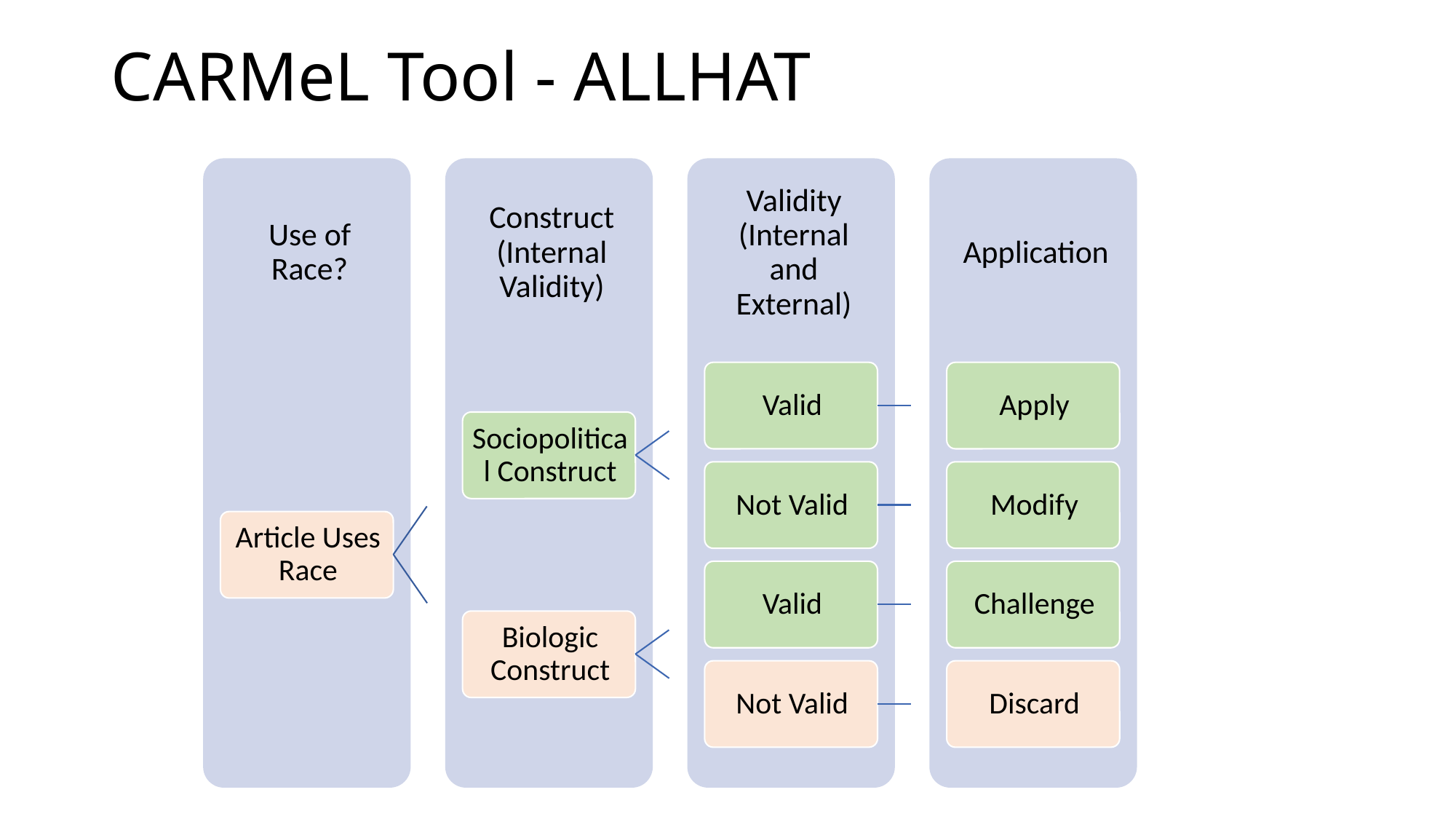

# CARMeL Tool - ALLHAT

## Slide 29
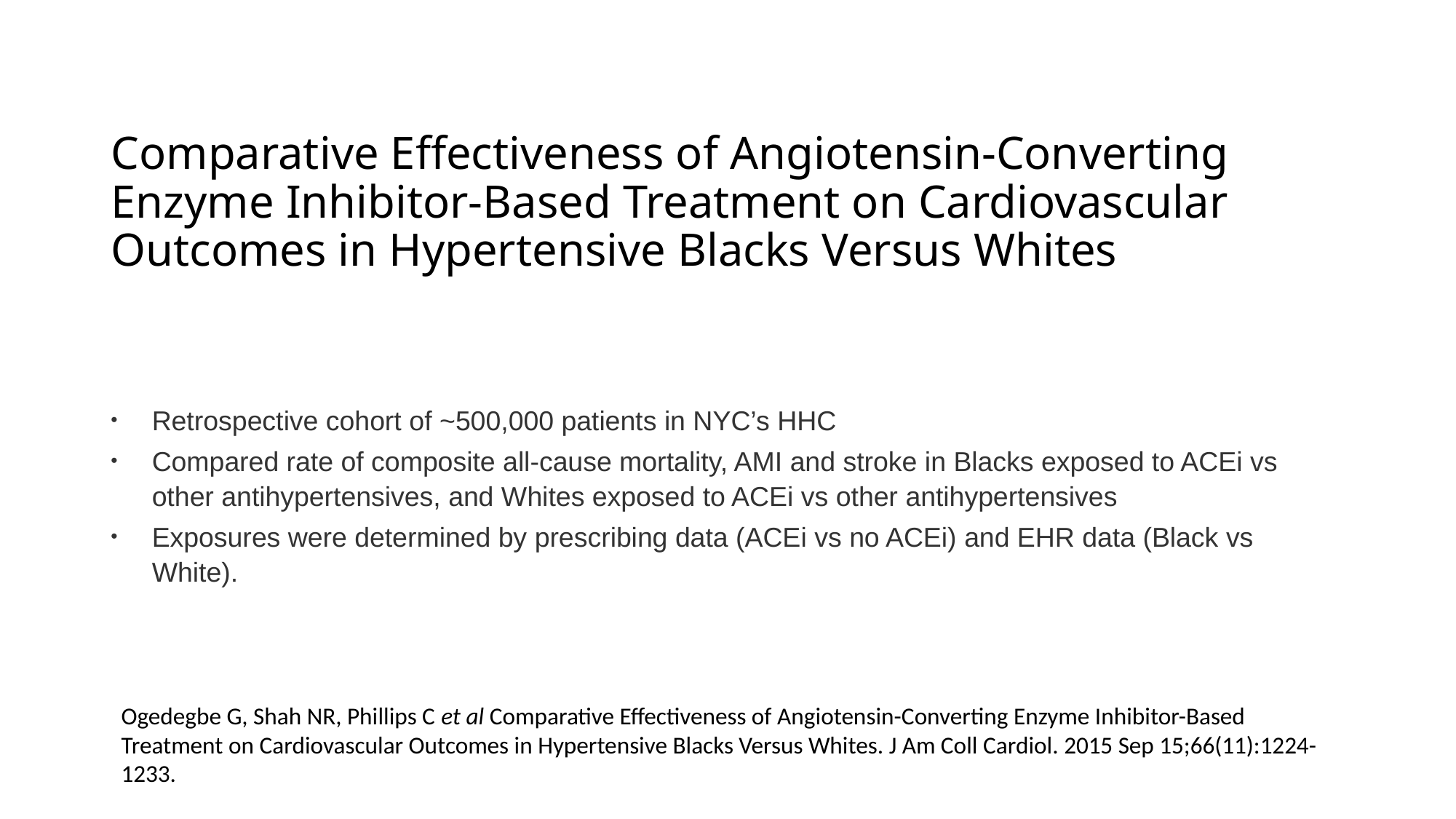

# Comparative Effectiveness of Angiotensin-Converting Enzyme Inhibitor-Based Treatment on Cardiovascular Outcomes in Hypertensive Blacks Versus Whites
Retrospective cohort of ~500,000 patients in NYC’s HHC
Compared rate of composite all-cause mortality, AMI and stroke in Blacks exposed to ACEi vs other antihypertensives, and Whites exposed to ACEi vs other antihypertensives
Exposures were determined by prescribing data (ACEi vs no ACEi) and EHR data (Black vs White).
Ogedegbe G, Shah NR, Phillips C et al Comparative Effectiveness of Angiotensin-Converting Enzyme Inhibitor-Based Treatment on Cardiovascular Outcomes in Hypertensive Blacks Versus Whites. J Am Coll Cardiol. 2015 Sep 15;66(11):1224-1233.

## Slide 30
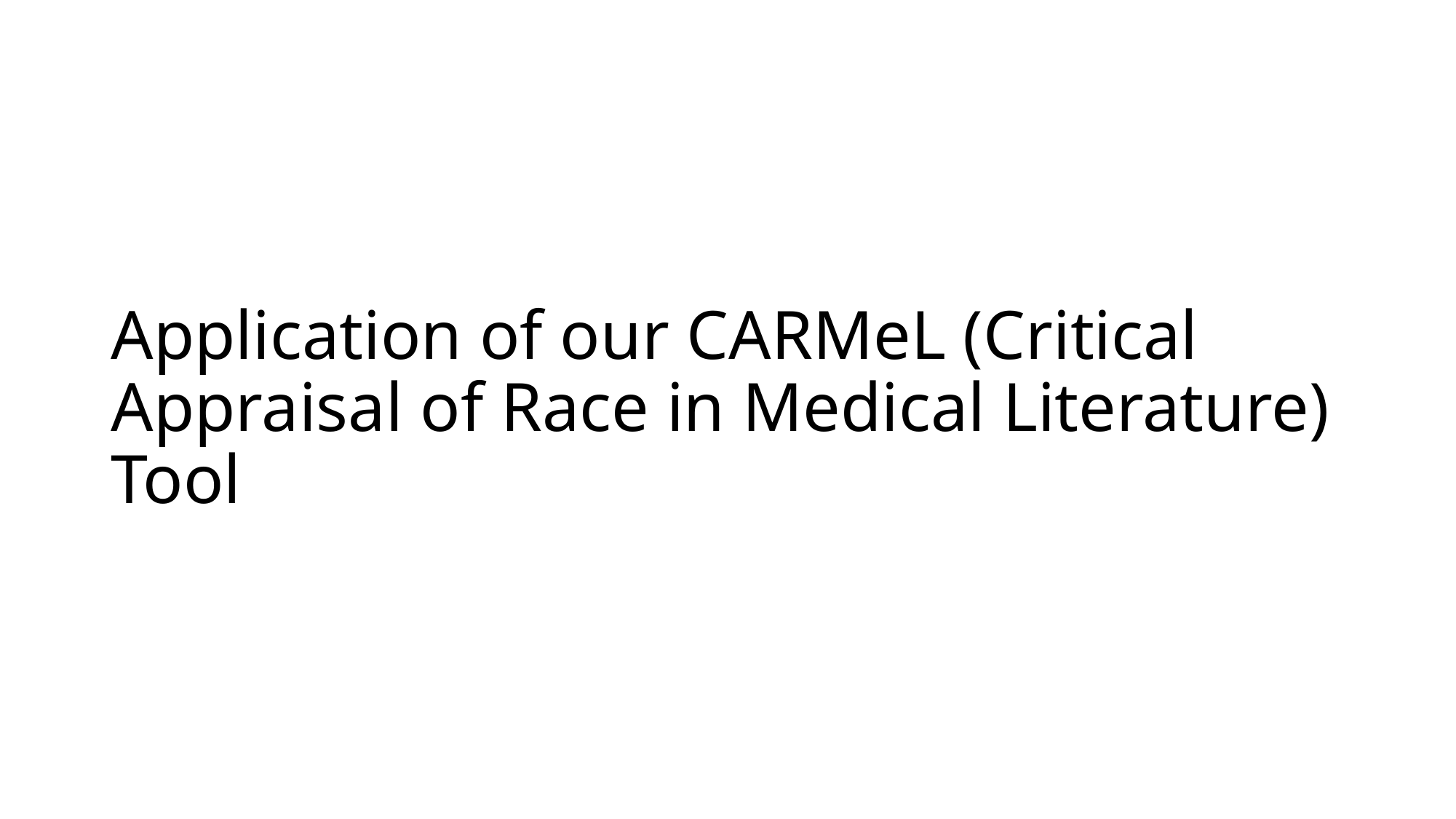

# Application of our CARMeL (Critical Appraisal of Race in Medical Literature) Tool

## Slide 31
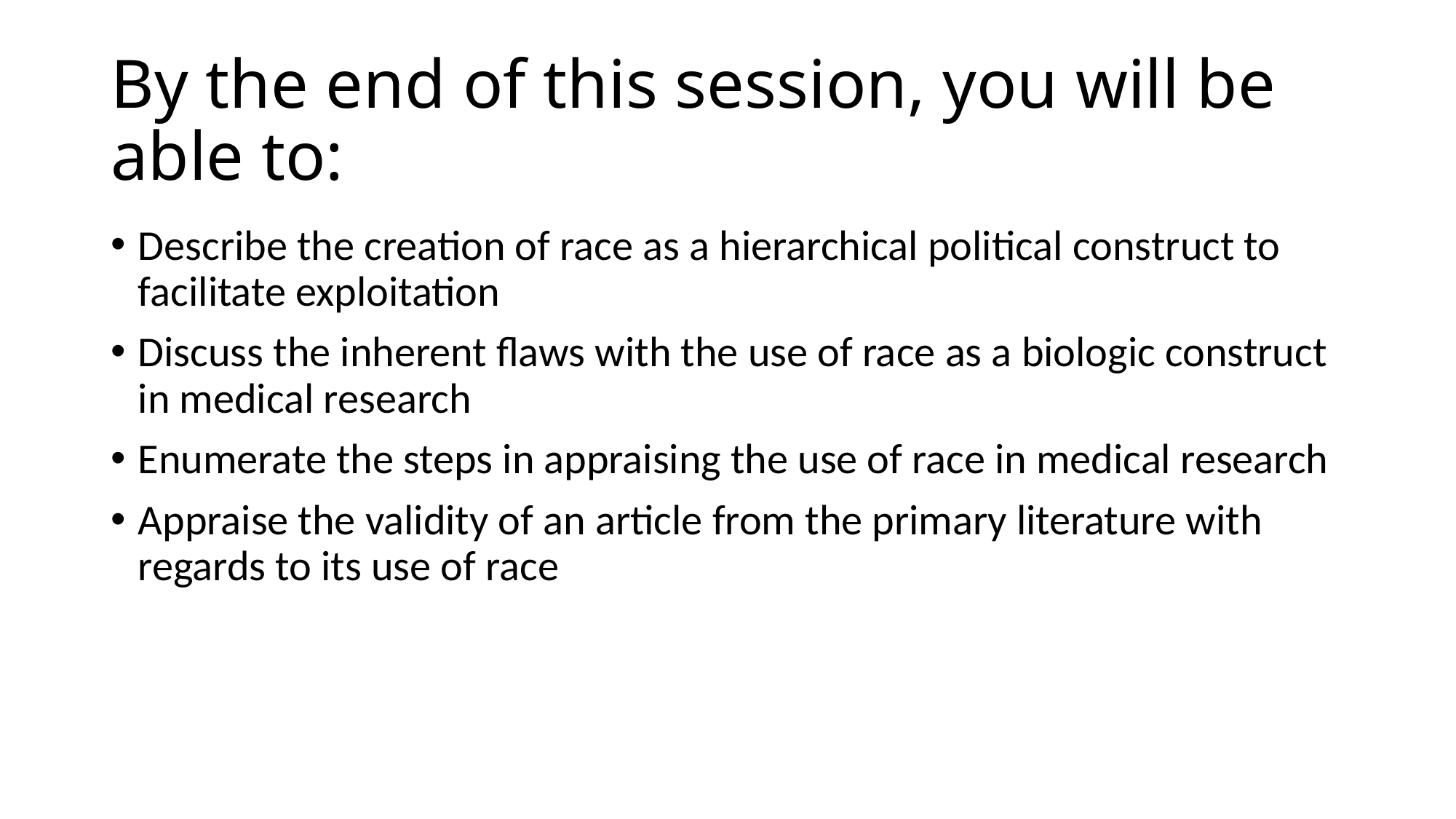

# By the end of this session, you will be able to:
Describe the creation of race as a hierarchical political construct to facilitate exploitation
Discuss the inherent flaws with the use of race as a biologic construct in medical research
Enumerate the steps in appraising the use of race in medical research
Appraise the validity of an article from the primary literature with regards to its use of race
