# Supplementary material for: From Race to Racism: Teaching a Tool to Critically Appraise the Use of Race in Medical Research
Source: MedEdPORTAL. 2022 Jan 24;18:11210. doi: 10.15766/mep_2374-8265.11210 (PMC8784584; doi:10.15766/mep_2374-8265.11210)
Supplement: Supplementary file 1 — CARMeL Tool.docxCARMeL Workshop.pptxFacilitator Guide.docxParticipant Guide.docxUME Postsession Assessment.docxGME Pre- and Postsession Survey.docx [file mep_2374-8265.11210-s001.zip › C. Facilitator Guide.docx]

A Tool to Critically Appraise the Use of Race in Medical Research

Facilitator’s Guide

**Session Overview and Objectives**

This workshop introduces learners to a framework they can use to appraise the use of race in medical research, with an emphasis on practicality and ease of use while reading articles in clinical practice. The sessions starts with an overview of concepts of race and ancestry, then explores some of the genetic and methodological flaws with the use of race in medical research as well as potential sociopolitical and clinical implications of the use of race as a biological construct. Finally, you will teach the Critical Appraisal of Race in Medical Literature (CARMeL) tool and practice applying this tool to an article from the primary literature.

By the end of this session, students will be able to:

1. Describe the historic creation of race as a hierarchical political construct.
2. Discuss the clinical and sociopolitical implications of the use of race as a biologic construct in medical research.
3. Enumerate the steps in appraising the use of race in clinical research.
4. Appraise the validity of an article with regards to its use of race.

**Preparing for the Session**

In order to prepare to facilitate this session we recommend you:

- Read this guide
- Review the slide set with accompanying facilitation notes
- Read the article to be appraised and the suggested appraisals found in this guide.

**Session Flow (for 90-minute session)**

| **Content** | **Suggested Time** | **Slides** | **Comments** |
| --- | --- | --- | --- |
| Introduction | 10 minutes | Slides 1-8 |  |
| Race and Ancestry | 10 minutes | Slides 9-14 | The anti-oppressive facilitation notes will be useful in this portion of the session |
| Ancestry and Genetics | 15 minutes | Slides 15-19 |  |
| Introduction of the CARMeL tool | 20 minutes | Slides 20-29 |  |
| Application of the CARMeL tool (break-out groups) | 30 minutes | Slide 30-31 and guide below | Slide 30: Use “Traditional Appraisal”  Slide 31: Use “Appraisal of Article’s Use of Race” |
| Wrap-up and Questions | 5 minutes | Slide 32 |  |

**Anti-Oppressive and Anti-Racist Facilitation Techniques (For use throughout the session)**

A central concept in anti-racist medical education is that there is no neutral space – clinical practices either increase racial inequities (are racist) or decrease such inequities (are anti-racist); educational practices either perpetuate racial inequities, including racist ideologies and internalized oppression in learners and oppressive power structures in educational spaces, or they disrupt such ideologies, systems and structures.

The forces that create and perpetuate such racist policies and practices are known as White Supremacy Culture. As White Supremacy is the cultural norm and ubiquitous in our society, it is present in every educational space, regardless of the content of the session, unless named and disrupted.

The techniques you use as a facilitator are either anti-racist and anti-oppressive, or racist and oppressive. Your goal as a facilitator should be to recognize White supremacy norms at play, and use anti-racist and anti-oppressive facilitation techniques to disrupt them. Sample techniques are presented below. We recommend also reviewing various online resources on this topic including those produced by Dismantling Racism Works (drWorks) and The Anti-Oppressive Resource and Training Alliance (AORTA).

| **White Supremacy Cultural Norm** | **Manifestation in Educational Space** | **Anti-Oppressive Facilitation Techniques** |
| --- | --- | --- |
| Power hoarding | Individuals with traditional power in society (White people, cis-gender men) speak more and are recognized more than others, interrupt others in the group, or re-state what others have said. | Use techniques such as “think-pair-share,” waiting 30 seconds prior to hearing response etc. to create space for those not being heard.  Name what you observe: “I have noticed that comments are often being restated by men before being acknowledged by the group.”  Synthesize comments/statements marginalized/oppressed voices in the room and ask those individuals if your synthesis is correct. |
| Right to comfort | Racist statements, including those that unintentionally perpetuate racist ideologies and scientific racism, go unchallenged, and are therefore assumed to be acceptable/true.  There is a fear of making individuals, particularly white men, uncomfortable. The burden of challenging racist ideas remains with members of the marginalized group. | Establish a culture of growth at the start of each session. This includes concepts of supporting individual growth while challenging ideas.  Name what you observe: “I have noticed that only people of color have shared their thoughts about racism in this case example.” |
| Supremacy of the written word | Published articles, textbooks and guidelines are viewed as the only or the primary source of knowledge and truth. Their conclusions are not challenged. Perspectives or marginalized and oppressed communities are not heard. | Intentionally include diverse perspectives (patients, communities).  Model thoughtful critique of bodies of scientific knowledge.  Challenge scientific racism, and “name those who do the writing.” |
| Either-or thinking | Participants are viewed as “either vocal or not participating.”  Participants struggle to identify racist policies and practices out of fear of “labeling someone/something racist.” Debate over “is this racist or not.”  Learners challenge the premise of the session. For example “I do not think these authors are racist – they are trying to find the best treatment for patients.” | Recognize that there are many forms of participating. Describe these at the start of a session and create opportunities for learners to participate.  Recognize that racism and oppression are ever-present and focus the discussion on consequences as opposed to intent of individual policies and practices.    As above, focus on consequences of the article/data – not the intent of the authors. One example: perpetuating false biological differences between races as a driver of cardiovascular inequities obscures the true drivers of these inequities. |

**Traditional Appraisal of the Article (For use on Slide 30)**

*Learners will NOT have read the article in advance*

- Before performing an appraisal of the article’s use of race, ask the participants to view the article as well as the participant guide, which includes the traditional appraisal below.
- Let participants know that this appraisal uses the traditional approach without any specific analysis of the use of race or its implications.
- Go through the below appraisal together, giving participants time to read the parts of the article called out in each question. They already have the answers, so the point is for them to familiarize themselves with the paper, the methods and the analysis.

*Traditional Appraisal of the Article*

**1) What is the study design? (Found in abstract, and in page 2 under Methods section)**

- Retrospective cohort involving nearly ½ a million patients in the HHC system.

**2) What is the primary outcome? Secondary outcomes? (Found in abstract, and in page 2 under Methods 🡪 Study Measures and Outcomes)**

- Primary: Composite of all-cause mortality, non-fatal acute MI and non-fatal stroke.
- Secondary: AMI, Stroke, All-cause mortality, hyperkalemia, hypokalemia, renal disease, CHF

**3) What were the exposure groups? Was the method for determining the exposure groups objectives and accurate? (Page 3 under Methods, also see page 7, study limitations)**

- Four exposure groups were created: Black-ACE, Black-No ACE, White-ACE, White-No ACE.
- ACE/No ACE was determined by prescription data, but unknown if prescriptions filled and medications taken
- Black vs White was based on EHR data (see below for critique of this approach).

**4) Were the groups adequately similar at the start of the trial? (Page 3 Statistical Analysis, Tables 1 and 2, and Page 4 first paragraph under Discussion)**

- No – there were significant differences in multiple variables. The authors state these were statistically corrected for other than VLDL, HDL and triglyceride levels.
- Because patients were not randomly assigned to treatment, “inverse probability of treatment weights” were applied to ensure that all treatment and non-treatment groups were balanced across potentially confounding covariates and minimize bias due to confounding by indication.

**5) Did the study adjust for important variables? (Page 3 Covariates used for risk adjustment)**

- Adjusted for age, sex, year of entry, # of clinic visits in previous year, baseline BPs, baseline creatinine, cholesterol, potassium, some medications and other BP meds, and used Charlson Comorbidity Index.

**6) Is it unlikely that there were unmeasured differences between the groups that may have affected the outcome? (Tables 1 and 2)**

- Based on a “traditional” appraisal no as most major medical factors that contribute to CVD were included. However can have additional discussion on this in the “Appraisal through the lens of race” section.

**7) Were all important outcomes considered?**

- Yes

**8) What is the hazard ratio for the primary outcome? (Table 3)**

*A hazard ratio is the rate at which events happen for one group compared to another. If the HR is 1, there is no difference between the rate at which at event happens between exposure groups. Similarly, if the 95% confidence interval crosses 1 there is no significant difference between the rate at which events occur between two different exposure groups.*

*The ratio of HRs compares two different HRs and describes likelihood of an event (similar to an odds ratio – does not calculate a rate).*

- The HR for composite of AMI, stroke and all-cause mortality was 1.11 (0.99-1.25 95% CI) for Blacks and 0.94 (0.84-1.06 95% CI) for Whites. In plane language, this means that the rate of composite AMI, stroke and all-cause mortality was 1.11x more likely in the ACEi group compared with the non-ACEi group (or, there is a 95% chance that the “real” HR is somewhere between 0.99 and 1.25), for Blacks. There was no difference between the ACEi exposure group and non-ACEi exposure group in Whites
- The Ratio of HRs for this composite was 1.18 (1.00-1.40 95% CI). This means that Blacks as compared to Whites were 1.18x more likely to have an event (AMI, stroke or all-cause mortality) when exposed to an ACEi vs other BP med. There is a 95% chance that the “real” ratio is somewhere between 1 and 1.4 x more likely.

**Appraisal of the Use of Article’s Use of Race (For use with slide 31)**

- Divide the learners into three groups
- Assign each group ONE of the appraisal domains (internal validity, external validity or applicability)
- Ask each group to answer the questions from the CARMeL tool that are included in their assigned domain. Let them know to select a representative from the group who will report back to the larger group after the breakout.
- Allow approximately 10 minutes for the break-out groups
- After 10 minutes and/or all groups are done, bring them back into one large group and ask each group to report back their findings.
- Facilitate a discussion using the completed table below.

| **Domain** | **Appraisal Questions** | **Sample Responses from Appraised Article** |
| --- | --- | --- |
| **Internal Validity** | Do the authors clearly define race?  If so how? Is this definition consistent throughout the data collection, analysis and discussion? | The authors do not pose a definition of race itself, other than how it is collected through the EHR as “typically” based on self-reporting. |
|  | To what extent does this article relay a biologic vs sociopolitical understanding of race? | The use of race throughout the article connotes an understanding of race as a biologic construct. The authors cite data that “blacks are inherently at higher risk for CV events” and state that they statistically controlled for “the inherent differences in CVD risk due to race (being black) in addition to controlling for baseline SBP.” |
|  | To what extent do the authors clearly define how data on race were collected and organized? | The authors describe that “In the EHR, patient race is typically recorded based on self-reporting, the gold standard for such classification. Race data were collapsed into African American or black, Caucasian or white, Asian, Native American/Alaskan, and Native Hawaiian/Other Pacific Islander, following the U.S. Office of Management and Budget classifications, with only blacks and whites included in the analysis.”  The actual methods used in the EHRs were not assessed.  The options for race included in the EHR are not discussed, and the methods by which these options were “collapsed” were not provided. |
|  | If applicable, were those who analyzed race blinded to the trial interventions? | Blinding was not discussed with regards to race. |
| **External Validity** | To what extent do the options for race collected, reported and analyzed in this paper reflect typical, contemporary racial identities, or the understanding of racial identity with my patient population? | The simplistic use of “African American or black” and “Caucasian or white” does not account for more complex racial identities commonly used by patients and communities. |
|  | Are racial categories missing and/or conflated? | Collapsing race data conflates multiple racial identities.  Only two classifications were analyzed, excluding many others. |
| **Applicability/Impact** | Are there significant biological, social, political or economic drivers of health that may be obscured conclusions made in this article? | Yes – multiple socio-political factors contribute to the inequities in cardiovascular health, including access to care, racial segregation and implicit bias. |
|  | In what ways does the use of race in this article contribute to dominant narratives? | The methods and discussion reinforce the concept of “Black” as a singular biologic race with intrinsic risk of hypertension, poor cardiovascular outcomes, and differential response to medications. |
| **Summary with Recommendation** | This article uses race as a biological construct, as evidenced by multiple references to “intrinsic risk” and equivalence of “black” patients across the United States. Significant threats to internal validity include an incomplete description of how race data were collected and collapsed, with no description of blinding amongst those collapsing and analyzing the data. Generalizability is challenging as only “black” and “white” races were analyzed. The application of these data may distract from broader and more potent drivers of inequities in cardiovascular health. Given the use of race as a biologic construct and significant methodological flaws in data collection and analysis, we recommend against the use of these data in patient care (see “green” pathway in the schematic to the right). | |

**Ask:** How might this article, within the context of JNC-8 guidelines and ALLHAT data, impact your clinical practice, if at all?

- Possible response: not at all

**Ask:** What factors do you/would you take into account when choosing an initial anti-hypertensive medication?

- Possible responses:
  - Possible secondary benefits (ACEi for patients with ischemic heart disease or diabetic microalbuminuria, beta blockers for patients with heart failure or palpitations/tachycardia)
  - Possible side effects: (beta blockers masking hypoglycemia in patients with diabetes, exacerbating COPD/asthma, worsening libido, exacerbating depression; ACEi worsening renal function/causing hyperkalemia (acutely); thiazide diuretics causing hyponatremia or worsening nocturia)
  - Cost
  - Dosing frequency
  - Patient prior experiences/perceptions
