# Supplementary material for: From Race to Racism: Teaching a Tool to Critically Appraise the Use of Race in Medical Research
Source: MedEdPORTAL. 2022 Jan 24;18:11210. doi: 10.15766/mep_2374-8265.11210 (PMC8784584; doi:10.15766/mep_2374-8265.11210)
Supplement: Supplementary file 1 — CARMeL Tool.docxCARMeL Workshop.pptxFacilitator Guide.docxParticipant Guide.docxUME Postsession Assessment.docxGME Pre- and Postsession Survey.docx [file mep_2374-8265.11210-s001.zip › D. Participant Guide.docx]

A Tool to Critically Appraise the Use of Race in Medical Research

Participant’s Guide

**Session Overview**

This workshop introduces you to the Critical Appraisal of Race in Medical Literature (CARMeL) tool. We will start with an overview of concepts of race and ancestry, then explore some of the genetic and methodological flaws with the use of race in medical research. We will explore potential sociopolitical and clinical implications of the use of race as a biological construct. Finally, you will learn about the CARMeL tool and practice applying this tool to an article from the primary literature.

This guide contains materials for use during the session and can serve as a resource when applying these skills to your clinical work. Your facilitator will instruct you when to refer to each part of the guide.

**Session Objectives**

By the end of this session, you will be able to:

1. Describe the historic creation of race as a hierarchical political construct.
2. Discuss the clinical and sociopolitical implications of the use of race as a biologic construct in medical research.
3. Enumerate the steps in appraising the use of race in clinical research.
4. Appraise the validity of an article with regards to its use of race.

**Sample Flaws, Challenges and Implication with the use of Race in Medical Research**

| **Genetic Flaws** | **Common Methodologic Flaws*** | **Clinical Challenges** | **Sociopolitical Implications** |
| --- | --- | --- | --- |
| Genetic variations is distributed continuously and overlaps between Old World Continental populations ^a^  More genetic  variation is found within Old World continental populations than between these populations^7^  Racial categories are sociopolitically created and vary over time and space ^b, c^ | Lack of consistent definitions of race and ancestry within and between publications  Lack of transparency in race data collection and interpretation  Conflation of racial categories | Practical challenges of reconciling complex individual ancestries with simplistic, often binary racial categories  Lack of training in discussing race and racism with patients | Positing intrinsic biological “risk” of illness obscures sociopolitical drivers of health inequities (e.g. racism)  Perpetuates illness burden on Black bodies  Connotes a human hierarchy |

*Methodological flaws may exist even when race is used as a socio-political construct

a) Jorde LB, Wooding SP. Genetic variation, classification and 'race'. Nat Genet. 2004 Nov;36(11 Suppl):S28-33. doi: 10.1038/ng1435. PMID: 15508000.

b) Yudell M, Roberts D, Desalle R, Tishkoff S. Taking Race Out of Human Genetics, Engaging a Century-Long Debate about the Role of Race in Science. Science. 2016 Feb: 564-565

c) Braun L, Fausto-Sterling A, Fullwiley D, Hammonds EM, Nelson A, Quivers W, Reverby SM, Shields AE. Racial categories in medical practice: how useful are they? PLoS Med. 2007 Sep;4(9):e271. doi: 10.1371/journal.pmed.0040271. PMID: 17896853; PMCID: PMC1989738.

**Critical Appraisal of Race in Medical Literature (CARMeL) Tool**

| **Domain** | **Appraisal Questions** |
| --- | --- |
| **Internal Validity** | Do the authors clearly define race?  If so how? Is this definition consistent throughout the data collection, analysis and discussion? |
|  | To what extent does this article relay a biologic versus sociopolitical understanding of race? |
|  | To what extent do the authors clearly define how data on race were collected and organized? |
|  | If applicable, were those who analyzed race blinded to the trial interventions? |
| **External Validity** | To what extent do the options for race collected, reported and analyzed in this paper reflect typical, contemporary racial identities, or the understanding of racial identity with my patient population? |
|  | Are racial categories missing and/or conflated? |
| **Applicability/Impact** | Are there significant social, political or economic drivers of health that may be obscured by conclusions made in this article? |
|  | In what ways does the use of race in this article contribute to dominant narratives? |
| **Summary with Recommendation** | 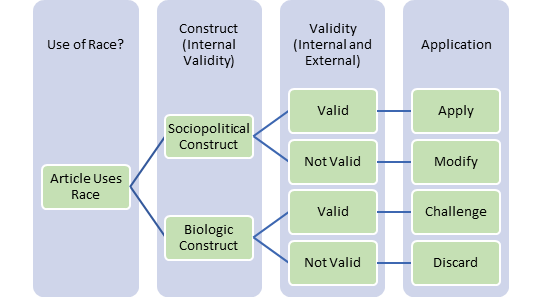**CHOOSE ONE**:  *APPLY*: Race is used as a sociopolitical construct with appropriate methods and no significant threats to internal or external validity. The sociopolitical implications are evaluated and the data is deemed appropriate to apply to patients or populations.  *MODIFY*: Race is used as a sociopolitical construct, but with some threats to internal or external validity. In this case appropriate observations are accepted, but due to methodologic flaws providers will need to modify the way they are applied.  *CHALLENGE:* Race is likely used as a biologic construct, but with few to little other threats to the internal validity of the study. These studies often yield accurate observations of racial inequities in health, but draw inaccurate conclusions regarding causality (i.e. posit racial differences in the prevalence of illness or response to treatment, as opposed to racism and oppression as the cause of the observations). The results of such studies can be used, however the political causes of the observed inequities must be attributed and dismantled. Examples include studies that observe true racial inequities in cardiovascular outcomes but attribute them to biological differences.  *DISCARD:*  Race is used as a biologic construct with significant threats to internal and/or external validity. The results should be challenged, and the conclusions not applied. This includes studies that suggest differential diagnostic tools, treatment algorithms or interventions based on biologic definitions of race, for example studies used to purport racial differences in glomerular filtration rate or expected lung function. |

**Traditional Appraisal of the Article (For use in workshop)**

**1) What is the study design? (Found in abstract, and in page 2 under Methods section)**

- Retrospective cohort involving nearly ½ a million patients in the HHC system.

**2) What is the primary outcome? Secondary outcomes? (Found in abstract, and in page 2 under Methods 🡪 Study Measures and Outcomes)**

- Primary: Composite of all-cause mortality, non-fatal acute MI and non-fatal stroke.
- Secondary: AMI, Stroke, All-cause mortality, hyperkalemia, hypokalemia, renal disease, CHF

**3) What were the exposure groups? Was the method for determining the exposure groups objectives and accurate? (Page 3 under Methods, also see page 7, study limitations)**

- Four exposure groups were created: Black-ACE, Black-NoACE, White-ACE, White-NoACE.
- ACE/NoACE was determined by prescription data, but unknown if prescriptions filled and medications taken
- Black vs White was based on EHR data (see below for critique of this approach).

**4) Were the groups adequately similar at the start of the trial? (Page 3 Statistical Analysis, Tables 1 and 2, and Page 4 first paragraph under Discussion)**

- No – there were significant differences in multiple variables. The authors state these were statistically corrected for other than VLDL, HDL and triglyceride levels.
- Because patients were not randomly assigned to treatment, “inverse probability of treatment weights” were applied to ensure that all treatment and non-treatment groups were balanced across potentially confounding covariates and minimize bias due to confounding by indication.

**5) Did the study adjust for important variables? (Page 3 Covariates used for risk adjustment)**

- Adjusted for age, sex, year of entry, # of clinic visits in previous year, baseline BPs, baseline creatinine, cholesterol, potassium, some medications and other BP meds, and used Charlson Comorbidity Index.

**6) Is it unlikely that there were unmeasured differences between the groups that may have affected the outcome? (Tables 1 and 2)**

- Based on a “traditional” appraisal no as most major medical factors that contribute to CVD were included. However can have additional discussion on this in the “Appraisal through the lens of race” section.

**7) Were all important outcomes considered?**

- Yes

**8) What is the hazard ratio for the primary outcome? (Table 3)**

*A hazard ratio is the rate at which events happen for one group compared to another. If the HR is 1, there is no difference between the rate at which at event happens between exposure groups. Similarly, if the 95% confidence interval crosses 1 there is no significant difference berween the rate at which events occur between two different exposure groups.*

*The ratio of HRs compares two different HRs, and describes likelihood of an event (similar to an odds ratio – does not calculate a rate).*

- The HR for composite of AMI, stroke and all-cause mortality was 1.11 (0.99-1.25 95% CI) for Blacks and 0.94 (0.84-1.06 95% CI) for Whites. In plane language, this means that the rate of composite AMI, stroke and all-cause mortality was 1.11x more likely in the ACEi group compared with the non-ACEi group (or, there is a 95% chance that the “real” HR is somewhere between 0.99 and 1.25), for Blacks. There was no difference between the ACEil exposure group and non-ACEi exposure group in Whites
- The Ratio of HRs for this composite was 1.18 (1.00-1.40 95% CI). This means that Blacks as compared to Whites were 1.18x more likely to have an event (AMI, stroke or all-cause mortality) when exposed to an ACEi vs other BP med. There is a 95% chance that the “real” ratio is somewhere between 1 and 1.4 x more likely.

**Appraisal of the Use of Article’s Use of Race (for use in break-out groups)**

| **Domain** | **Appraisal Questions** | **Responses from Appraised Article** |
| --- | --- | --- |
| **Internal Validity** | Do the authors clearly define race?  If so how? Is this definition consistent throughout the data collection, analysis and discussion? |  |
|  | To what extent does this article relay a biologic vs sociopolitical understanding of race? |  |
|  | To what extent do the authors clearly define how data on race were collected and organized? |  |
|  | If applicable, were those who analyzed race blinded to the trial interventions? |  |
| **External Validity** | To what extent do the options for race collected, reported and analyzed in this paper reflect typical, contemporary racial identities, or the understanding of racial identity with my patient population? |  |
|  | Are racial categories missing and/or conflated? |  |
| **Applicability/Impact** | Are there significant biological, social, political or economic drivers of health that may be obscured conclusions made in this article? |  |
|  | In what ways does the use of race in this article contribute to dominant narratives? |  |
