# Supplementary material for: From Race to Racism: Teaching a Tool to Critically Appraise the Use of Race in Medical Research
Source: MedEdPORTAL. 2022 Jan 24;18:11210. doi: 10.15766/mep_2374-8265.11210 (PMC8784584; doi:10.15766/mep_2374-8265.11210)
Supplement: Supplementary file 1 — CARMeL Tool.docxCARMeL Workshop.pptxFacilitator Guide.docxParticipant Guide.docxUME Postsession Assessment.docxGME Pre- and Postsession Survey.docx [file mep_2374-8265.11210-s001.zip › Educational Summary Report_11210.pdf]

MedEdPORTAL® | The AAMC Journal of Teaching and Learning Resources

Original Publication Open Access

From Race to Racism: Teaching a Tool to Critically Appraise the Use of Race in Medical Research

Amy Garvey, MD, Giselle Lynch, MD, Mayce Mansour, MD, Andrew Coyle, MD, Sabrina Gard, MD, MPH, Joseph Truglio, MD, MPH*

*Corresponding author: joseph.truglio@mssm.edu

Abstract

Introduction: Despite the ubiquitous use of race within scientific literature, medical trainees are not taught how to critically appraise the use of racial categories. We developed a tool to appraise the use of race in medical literature and a workshop to teach this approach. **Methods:** Third-year medical students and second- and third-year residents participated in workshops between 2015 and 2018. We evaluated our UME workshop with a postworkshop survey. We evaluated our GME workshop with a pretest, immediate posttest, and 6-month posttest on self-assessed knowledge, skills, and use of the Critical Appraisal of Race in Medical Literature (CARMeL) tool in subsequent journal clubs. **Results:** We delivered this workshop to 560 students and 82 residents. Of the initial 140-student cohort evaluating the workshop, 99 (71% response rate) highly rated clarity of presentation, quality of teaching, and quality of slides. Of PGY 2 and PGY 3 residents, 67 (82% response rate) rated the workshop greater than 4.5 out of 5 on quality, clarity, and appropriateness of content. Residents had significant improvements in self-assessed knowledge and skills immediately after the session and 6 months later. Of residents, 74% reported using the CARMeL tool in subsequent presentations. **Discussion:** We designed the CARMeL tool and a workshop to teach it. Trainees rated this workshop as useful, with the majority of residents later applying the tool. Limitations included a lack of objective assessment of knowledge acquisition. We recommend that institutions invest time in faculty development and pair new faculty with those experienced in anti-oppressive facilitation.

Keywords
Racism, Anti-Racism, Evidence-Based Medicine, Diversity, Inclusion, Heath Equity

Educational Objectives

By the end of this session, learners will be able to:

1. Describe the historic creation of race as a hierarchical political construct.
2. Discuss the clinical and sociopolitical implications of the use of race as a biologic construct in medical research.
3. Enumerate the steps in appraising the use of race in clinical research.
4. Appraise the validity of an article with regard to its use of race.

Citation:
Garvey A, Lynch G, Mansour M, Coyle A, Gard S, Truglio J. From race to racism: teaching a tool to critically appraise the use of race in medical research. *MedEdPORTAL*. 2022;18:11210.
https://doi.org/10.15766/mep_2374-8265.11210

Introduction

Racial health inequities, produced by racist policies and practices, are pervasive in the United States. Social scientists and race scholars often define race as a sociopolitical system of categorizing and stratifying people based on arbitrary phenotypes for the purpose of exploitation. Clearly stated by Professor Dorothy Roberts, "Race is the product of racism; racism is not the product of race."[1] In contrast, biomedical research often utilizes race as a biologic reality rooted in specious genetic differences. Many treatments, diagnostic modalities, and clinical prediction tools employ racial categories as distinct, fixed, and easily identifiable, reinforcing ideas of racially distinct mechanisms of illness.[2] This leads to the differential treatment of patients based on racial classifications and obscures the true driver of racial inequities: racism. Common examples include studies and clinical practices that accurately identify racial inequities but attribute these inequities to inherent, biological racial differences (e.g., risk of atherosclerotic cardiovascular disease, colon cancer) and measurements and interventions based on inaccurate racial differences in

Copyright © 2022 Garvey et al. This is an open-access publication distributed under the terms of the Creative Commons Attribution license.

physiologic characteristics or responses to pharmacologic therapy (e.g., estimated glomerular filtration rate, predicted lung volumes, predicted responses to various cardiovascular medications). Such studies and frameworks that view race as a biologic construct are recognized as flawed by impacted communities, community organizers, scholars, and professional organizations.[1-6]

Despite the ubiquitous use of racial categories within scientific literature and clinical medicine, medical students and residents are not routinely taught how to critically appraise the use of race within medical literature. Due to this lack of skills-based training, trainees are often ill prepared to distinguish appropriate from inappropriate use of race and are thus unable to effectively address issues related to race and racism in journal clubs and clinical practice. The result is a conflation of sociopolitical and biologic constructs of race leading to misinformed practices and poorer outcomes for patients of color, in particular Black patients. This is further reinforced through hospital-wide use of race-based medical tools and contemporary literature in high impact factor journals that continue to use race as a primary variable in their results.[7]

The past decade has seen a renewed call for improved education around issues of racism, bias, and structural determinants of health, largely driven by Black health activists, students, and trainees.[8,9] Because of the Black Lives Matter movement, racism, in particular anti-Black racism, has taken center stage in the current national dialogue. Many academic institutions now have programs and initiatives to address racism, with an emphasis on inequities. To our knowledge, none specifically address the critical appraisal of race in medical literature. A review of *MedEdPORTAL* revealed 46 resources related to race or racism but no currently available resource describing a tool for appraising the use of race in the primary medical literature.

To address this clinical and educational need, we developed the Critical Appraisal of Race in Medical Literature (CARMeL) tool (Appendix A) to help learners assess the use of race in medical research and an interactive workshop to teach this tool to rising third-year medical students and senior internal medicine residents.

Methods

We developed the CARMeL tool in 2015 using an iterative process examining multiple articles in a wide variety of disciplines for their use of race as a variable. In consultation with specialists in these fields as well as social scientists, we designed questions that assessed for the most common and most significant methodological flaws with the use of race. We organized these questions to align with the common *internal validity, external validity, applicability* evidence-based medicine (EBM) framework while also being concise enough for routine use in academic journal clubs and day-to-day clinical practice. The goal was for users to be able to distinguish if an article used a biological or sociopolitical framework for the category of race while also appraising the methods used to evaluate race as a variable. The end result of this appraisal was a recommendation to apply, modify, challenge, or discard/not use the conclusions or recommendations of the appraised article.

We designed a workshop to teach the CARMeL tool as part of the existing UME and GME EBM curricula. The preexisting EBM curricula included a series of workshops that followed the *ask, acquire, appraise, and apply* framework for UME students and a weekly journal club for GME residents.[10] Within this frequently used EBM framework, an individual first asks a clinical question, then turns to the medical literature to acquire data to answer this question, appraises the validity of these data, and finally applies the data to the patient or population at hand. We anchored the workshop content around the appraise step of traditional EBM curricula and structured the session using a standard journal club model. In a traditional appraisal of an article, trainees would consider the study's internal validity (the extent to which study design increases or decreases the risk of bias), external validity (the extent to which the study design promotes generalizability of findings), and applicability (the ability to apply the results of a study to one particular patient or clinical scenario). Our tool asks similar questions of an article's validity and applicability but is specific to the article's use of race.

Setting

UME and GME learners had independent sessions. UME learners were rising third-year students who participated in this workshop as a mandatory session during a preclerkship clinical skills week. At our institution, rising third-year students had not yet participated in any clinical clerkships but had some longitudinal clinical experiences as part of their doctoring course. GME learners were second- and third-year internal medicine residents and participated in this workshop as a mandatory part of their longitudinal ambulatory care didactic series. Both groups had received prior sessions on racism and bias (UME) or social determinants of health highlighting racial inequalities (GME). The content of the workshop did not differ between the two cohorts.

Implementation

Each session included groups of 15-20 learners with one facilitator. Facilitators were senior medical students, internal

Copyright © 2022 Garvey et al. This is an open-access publication distributed under the terms of the Creative Commons Attribution license.

medicine residents, and faculty chosen based on knowledge of the subject matter and/or willingness to engage with the material. We provided all facilitators with a copy of the CARMeL tool (Appendix A), the slides with facilitation notes (Appendix B), and a facilitator's guide (Appendix C) at least 1 week prior to the session. We also held a 1-hour facilitator development training prior to each session, which was necessary to ensure all facilitators were adequately prepared for the session given differences in educational background and experience. During the development session, one of the workshop developers reviewed the facilitation guide, provided training on anti-oppression facilitation techniques, and answered facilitators' questions.

We also provided all participants with a participant's guide (Appendix D) prior to the session. The workshops started with a discussion of race as a sociopolitical construct, including common methodological pitfalls of the use of race in medical research. Facilitators then presented the CARMeL tool. This tool considers the use of race from the lens of internal validity (e.g., is race being used as a biologic or sociopolitical construct? How was race data collected in the study?) and external validity (e.g., are the racial categories used generalizable to other studies, and are racial classifications pertinent to our patient populations?), with the final step centering on applicability to patients and the societal implications of the application of these results.

Facilitators first modeled this novel approach using the Antihypertensive and Lipid-Lowering Treatment to Prevent Heart Attack Trial (ALLHAT) as an example.[11] The ALLHAT was an 8-year, randomized, double-anonymized study that compared the use of different antihypertensive regimens on a range of cardiovascular disease outcomes. We selected this trial due to its impact, as it is the largest clinical trial thus far on antihypertensive medication, and its immediate influence on patient care through the Eighth Joint National Committee guidelines for the management of high blood pressure in adults.[12] Participants next reviewed an article from the medical literature (investigating cardiovascular outcomes with different antihypertensive agents, with race being used as a variable) and read a traditional appraisal from an EBM perspective.[13] Participants then worked in pairs to apply the framework using this guide and reported back to the larger group for discussion. The session concluded with a reflection of how these skills could be applied in clinical practice.

Evaluation

We used course evaluations (Appendix E) to evaluate perceived effectiveness of our UME workshop with postsurvey analyses of the clarity and quality of teaching. We evaluated our GME workshop with a pretest, immediate posttest, and 6-month follow-up surveys, as well as self-assessment of PGY 3 residents' EBM presentations (Appendix F). We did not evaluate the impact of individual facilitator factors (student vs. faculty, number of prior sessions taught, etc.) on learning. All internal medicine residents were required to lead one journal club discussion in the residency didactic curriculum throughout the year, and at the end of the workshop, facilitators encouraged residents to incorporate the CARMeL tool into their teaching. Faculty observed resident-led journal club discussions following the workshop to assess the incorporation of this tool.

We used SPSS statistical software to analyze the GME surveys, with effect sizes calculated by Cohen's *d*. The Icahn School of Medicine at Mount Sinai Institutional Review Board deemed further review of this project not necessary.

Results

During our evaluation phase, we delivered this workshop to 560 medical students between 2015 and 2018 and to 82 internal medicine residents during the 2018-2019 academic year. We taught the UME cohorts via 12-14 simultaneous small groups during a single session once per year. We taught the GME cohorts in four separate small-group sessions during four consecutive ambulatory care blocks each year. Due to differences in curricular structure between the two cohorts (individual course in UME vs. longitudinal curriculum in GME), we used different evaluation methods for each cohort.

UME

We conducted anonymous course evaluations for the initial 140-student cohort (2015) using a 5-point Likert scale, (1 = *unacceptable*, 5 = *excellent*). Mean scores for clarity of presentation, quality of teaching, and quality of slides were 4.3, 4.4, and 4.5, respectively, with 99 out of 140 participants responding (71% response rate).

GME

We distributed anonymous surveys before, immediately after, and 6 months after the GME workshops during the 2018-2019 academic year. These surveys assessed residents' satisfaction with the workshop, self-assessed knowledge and confidence, perceived barriers to implementing material, and self-reported behavior, with scores rated on a 5-point Likert scale (1 = *completely disagree*, 5 = *completely agree*). All 82 eligible PGY 2 and PGY 3 residents in the internal medicine residency program participated in the workshop in 2018, with 67 of 82

Copyright © 2022 Garvey et al. This is an open-access publication distributed under the terms of the Creative Commons Attribution license.

completing the pretest (82% response rate), 67 of 82 completing the immediate posttest (82% response rate), and 65 of 82 completing the 6-month posttest (79% response rate), for an overall response rate of 81% (199 of 246). Residents rated the workshop on the immediate posttest as 4.6 out of 5 for quality, 4.5 out of 5 for clarity, 4.5 out of 5 for appropriate amount of content, and 4.4 out of 5 for likelihood of changing their practice.

Resident responses demonstrated significant improvements in perceived knowledge and confidence, with retention of this improvement over a 6-month time frame (Table). At baseline, residents reported low levels of confidence in their ability to differentiate concepts of race and ancestry as well as appraise an article's use of race (the average scores were below 3, the *neutral* response). Residents had significant improvements in self-assessed knowledge and skills, with statistically significant improvements noted immediately after the session ($p < .01$) and 6 months later ($p < .01$), with large effect sizes. Effect sizes were calculated by Cohen's d, with effect sizes considered small ($0.20 \leq d \leq 0.49$), medium ($0.50 \leq d \leq 0.79$), or large ($d \geq 0.80$). There were no statistically significant differences in their assessment immediately after the session and 6 months later, demonstrating perceived retention of the material. Furthermore, of the 47 residents who led their journal club presentation after the workshop, 35 (74%) reported using the CARMeL tool to assess the use of race in the article they presented to the group.

Discussion

We designed a tool for UME and GME learners to critically examine the use of race in medical literature and a workshop that allowed learners to practice applying this structured framework. UME participants rated the session highly in both clarity and quality, and GME participants' self-assessed knowledge and skills significantly improved over a prolonged period. We now teach this session annually in the UME curriculum and, due to ongoing positive feedback from students, have included a second session appraising additional articles.

A major strength of this resource is our novel CARMeL tool, which gives trainees a systematic approach to consider the use of race in medical studies and is generalizable to medical literature across disciplines. Subsequent workshops in the UME and GME setting have successfully applied this tool to articles and guidelines regarding colorectal cancer screening,[14] the use of long-acting beta agonists in the management of asthma,[15-17] and the management of hyperbilirubinemia in newborns.[18,19] We have also delivered this workshop at a national conference and by invitation at multiple medical schools and residency program conferences around the country. In some cases, we have observed successful facilitation of this workshop by local faculty at other institutions. While not a formal evaluation, these experiences and observations suggest the generalizability of the CARMeL tool and the workshop.

Limitations include a lack of objective assessment of knowledge acquisition and a lack of assessment of the quality of application of this tool in subsequent EBM sessions by trainees. Evaluating the extent to which residents effectively utilize this framework in EBM presentations and in day-to-day clinical practice would better assess the application of the skills taught in this workshop. A major barrier to this assessment is faculty capacity to effectively evaluate such application in real time to a wide variety of articles. To meet this need, we are developing and validating faculty tools and enhanced faculty development so that the application of this tool can be more broadly and more accurately assessed. An additional limitation is a difference in evaluation methods between our UME and GME cohorts. This is due to differences in overall curricular structure and availability of students for follow-up surveys and observations. Residents have a more uniform curricular schedule and universally participate in follow-up journal

Table. Results of Pre- and Postssession GME Surveys

Statement[a]	Presession[b]	Immediate Postsession[c]	6-Month Postsession[d]	Presession to Immediate Postsession Change (*p*, Effect Size)	Presession to 6-Month Postsession Change (*p*, Effect Size)	Immediate Postsession to 6-Month Postsession Change (*p*, Effect Size)
I can differentiate concepts of race and ancestry as they apply to clinical practice.	3.1	4.0	4.0	0.9 (<.01, 1.4)	0.8 (<.01, 1.3)	0.0 (.80, 0.1)
I can describe common flaws with the use of race as a biologic construct.	3.1	4.2	4.1	1.1 (<.01, 1.6)	1.0 (<.01, 1.4)	−0.1 (.43, 0.2)
I am able to appraise an article's use of race.	2.8	4.1	4.0	1.3 (<.01, 2.3)	1.2 (<.01, 1.8)	−0.1 (.30, 0.3)

[a]Participants rated each statement on a 5-point Likert scale (1 = *completely disagree*, 5 = *completely agree*).
[b]Sixty-seven participants completed the presession survey.
[c]Sixty-seven participants completed the immediate postsession survey.
[d]Sixty-five participants completed the 6-month postsession survey.

Copyright © 2022 Garvey et al. This is an open-access publication distributed under the terms of the Creative Commons Attribution license.

clubs with faculty trained on the use of our tool. Students enter their clerkship rotations with variable schedules and inconsistent availability of faculty trained in the use of our tool. As we more uniformly integrate this content into the clerkships, we hope to assess the use of the CARMeL tool by students in the clinical environment.

We learned several lessons in developing and implementing this workshop. Faculty need significant development not only regarding workshop content but also in anti-oppressive facilitation techniques. Such techniques are included in the facilitator's guide (Appendix C). After each session, we held informal faculty debriefs. This allowed us to collect frequently asked questions and challenging topics of discussion, which are also included in the facilitator's guide. We recommend that institutions hoping to use this resource invest significant time in faculty development and consider pairing new faculty with those experienced in anti-racist and anti-oppressive facilitation techniques. We also recommend adjusting the emphasis and time allocation within the sessions to meet the needs of the learners based on their prior exposure to similar content. Our UME and GME cohorts had different levels of prior formal anti-racism content. Faculty experience from teaching this workshop at our institution, national conferences, and other medical schools suggests that while the workshop can be given alone, learners benefit from prior and sustained exposure to anti-racist medical training. It is particularly helpful for learners to be familiar with the fact that race is a sociopolitical, rather than biological, invention, which is covered in this workshop. For audiences less familiar with these concepts, we suggest allocating additional time to the first portion of the workshop discussing the creation of race and flaws inherent to biologic frameworks of race.

The work of Black activists, including medical students, sociologists, and medical historians, hastened many medical trainees and practitioners to evaluate the role of medicine in the perpetuation of structural racism. The misuse of race as a biologic construct in medicine leads to differential treatment of our patients and exacerbates inequities in care. Without the tools to critically appraise the use of race in medical research, trainees and clinicians are likely to perpetuate specious beliefs of biological differences between sociopolitical racial groups, obscuring true drivers of racial health inequities and missing opportunities to identify and change health policies and clinical practices that negatively impact their patients. By addressing the use of race in medical literature, we hope to help our trainees build a foundation by which they can dismantle racist scientific practice throughout their careers.

Appendices

A. CARMeL Tool.docx
B. CARMeL Workshop.pptx
C. Facilitator Guide.docx
D. Participant Guide.docx
E. UME Postsession Assessment.docx
F. GME Pre- and Postsession Survey.docx

All appendices are peer reviewed as integral parts of the Original Publication.

Amy Garvey, MD: Third-Year Resident, Department of Radiology and Imaging Sciences, Emory University School of Medicine

Giselle Lynch, MD: Third-Year Resident, Department of Ophthalmology, Icahn School of Medicine at Mount Sinai

Mayce Mansour, MD: Assistant Professor, Department of Medicine and Department of Medical Education, Icahn School of Medicine at Mount Sinai

Andrew Coyle, MD: Associate Professor, Department of Internal Medicine, University of Wisconsin School of Medicine and Public Health

Sabrina Gard, MD, MPH: Assistant Professor, Department of Medicine, Icahn School of Medicine at Mount Sinai

Joseph Truglio, MD, MPH: Assistant Professor, Department of Medicine, Department of Pediatrics, and Department of Medical Education, Icahn School of Medicine at Mount Sinai; ORCID: https://orcid.org/0000-0002-8718-6092

Acknowledgments
We would like to acknowledge the many students who inspired this work, in particular the students of the Anti-Racism Coalition (ARC) at the Icahn School of Medicine at Mount Sinai. We also thank Dr. Ann-Gel Palermo and Dr. David Muller for their support.

Disclosures
None to report.

Funding/Support
None to report.

Prior Presentations
Lynch G, Garvey A, Palermo AGS, Truglio J, Muller D. Critically appraising the use of race in biomedical research: a novel educational approach. Presented at: Society for General Internal Medicine National Meeting; April 11-14, 2018; Denver, CO.

Ethical Approval
The Icahn School of Medicine at Mount Sinai Institutional Review Board deemed further review of this project not necessary.

Copyright © 2022 Garvey et al. This is an open-access publication distributed under the terms of the Creative Commons Attribution license.

References

1. Roberts D. *Fatal Invention: How Science, Politics, and Big Business Re-create Race in the Twenty-First Century*. The New Press; 2011.
2. Vyas DA, Eisenstein LG, Jones DS. Hidden in plain sight—reconsidering the use of race correction in clinical algorithms. *N Engl J Med*. 2020;383(9):874-882. https://doi.org/10.1056/NEJMms2004740
3. Braun L. *Breathing Race Into the Machine: The Surprising Career of the Spirometer From Plantation to Genetics*. University of Minnesota Press; 2014. https://doi.org/10.5749/minnesota/9780816683574.001.0001
4. Establishing a task force to reassess the inclusion of race in diagnosing kidney diseases. National Kidney Foundation. July 2, 2020. Accessed August 25, 2021. https://www.kidney.org/news/establishing-task-force-to-reassess-inclusion-race-diagnosing-kidney-diseases
5. Yudell M, Roberts D, DeSalle R, Tishkoff S. Taking race out of human genetics. *Science*. 2016;351(6273):564-565. https://doi.org/10.1126/science.aac4951
6. Braun L, Fausto-Sterling A, Fullwiley D, et al. Racial categories in medical practice: how useful are they? *PLoS Med*. 2007;4(9):e271. https://doi.org/10.1371/journal.pmed.0040271
7. Ojji DB, Mayosi B, Francis V, et al; CREOLE Study Investigators. Comparison of dual therapies for lowering blood pressure in Black Africans. *N Engl J Med*. 2019;380(25):2429-2439. https://doi.org/10.1056/NEJMoa1901113
8. Acosta D, Ackerman-Barger K. Breaking the silence: time to talk about race and racism. *Acad Med*. 2017;92(3):285-288. https://doi.org/10.1097/ACM.0000000000001416
9. Wear D, Zarconi J, Aultman JM, Chyatte MR, Kumagai AK. Remembering Freddie Gray: medical education for social justice. *Acad Med*. 2017;92(3):312-317. https://doi.org/10.1097/ACM.0000000000001355
10. Guyatt G, Rennie D, Meade MO, Cook DJ, eds. *Users' Guides to the Medical Literature: A Manual for Evidence-Based Clinical Practice*. 3rd ed. McGraw-Hill Education; 2015.
11. ALLHAT Officers and Coordinators for the ALLHAT Collaborative Research Group. Major outcomes in high-risk hypertensive patients randomized to angiotensin-converting enzyme inhibitor or calcium channel blocker vs diuretic: the Antihypertensive and Lipid-Lowering Treatment to Prevent Heart Attack Trial (ALLHAT). *JAMA*. 2002;288(23):2981-2997. https://doi.org/10.1001/jama.288.23.2981
12. James PA, Oparil S, Carter BL, et al. 2014 evidence-based guideline for the management of high blood pressure in adults: report from the panel members appointed to the Eighth Joint National Committee (JNC 8). *JAMA*. 2014;311(5):507-520. https://doi.org/10.1001/jama.2013.284427
13. Ogedegbe G, Shah NR, Phillips C, et al. Comparative effectiveness of angiotensin-converting enzyme inhibitor-based treatment on cardiovascular outcomes in hypertensive Blacks versus Whites. *J Am Coll Cardiol*. 2015;66(11):1224-1233. https://doi.org/10.1016/j.jacc.2015.07.021
14. Rex DK, Khan AM, Shah P, Newton J, Cummings OW. Screening colonoscopy in asymptomatic average-risk African Americans. *Gastrointest Endosc*. 2000;51(5):524-527. https://doi.org/10.1016/S0016-5107(00)70283-5
15. Wechsler ME, Yawn BP, Fuhlbrigge AL, et al; BELT Investigators. Anticholinergic vs long-acting β-agonist in combination with inhaled corticosteroids in Black adults with asthma: the BELT randomized clinical trial. *JAMA*. 2015;314(16):1720-1730. https://doi.org/10.1001/jama.2015.13277
16. Sears MR. The Salmeterol Multicenter Asthma Research Trial. Letter to the editor. *Chest*. 2006;130(3):928. https://doi.org/10.1378/chest.130.3.928
17. Nelson HS, Dorinsky PM. Reply to Sears. *Chest*. 2006;130(3):928-929.
18. Newman TB, Xiong B, Gonzales VM, Escobar GJ. Prediction and prevention of extreme neonatal hyperbilirubinemia in a mature health maintenance organization. *Arch Pediatr Adolesc Med*. 2000;154(11):1140-1147. https://doi.org/10.1001/archpedi.154.11.1140
19. Newman TB, Escobar GJ. Letter to the editor. *Ambul Pediatr*. 2001;1(2):126.

Received: April 3, 2021
Accepted: October 5, 2021
Published: January 24, 2022

Copyright © 2022 Garvey et al. This is an open-access publication distributed under the terms of the Creative Commons Attribution license.
